# Properties and Acceleration Mechanisms of Electrons Up To 200 keV Associated With a Flux Rope Pair and Reconnection X‐Lines Around It in Earth's Plasma Sheet

**DOI:** 10.1029/2022JA030721

**Published:** 2022-12-23

**Authors:** Weijie Sun, Drew L. Turner, Qile Zhang, Shan Wang, Jan Egedal, Trevor Leonard, James A. Slavin, Qiang Hu, Ian J. Cohen, Kevin Genestreti, Gangkai Poh, Daniel J. Gershman, Andrew Smith, Guan Le, Rumi Nakamura, Barbara L. Giles, Robert E. Ergun, James L. Burch

**Affiliations:** ^1^ Department of Climate and Space Sciences and Engineering University of Michigan Ann Arbor MI USA; ^2^ Space Exploration Sector Johns Hopkins Applied Physics Laboratory Laurel MD USA; ^3^ Los Alamos National Laboratory Los Alamos NM USA; ^4^ Department of Astronomy University of Maryland College Park MD USA; ^5^ Department of Physics University of Wisconsin‐Madison Madison WI USA; ^6^ Laboratory for Atmospheric and Space Physics University of Colorado Boulder Boulder CO USA; ^7^ Department of Space Science Center for Space Plasma and Aeronomic Research The University of Alabama in Huntsville Huntsville AL USA; ^8^ Southwest Research Institute San Antonio TX USA; ^9^ NASA Goddard Space Flight Center Greenbelt MD USA; ^10^ Center for Research and Exploration in Space Sciences and Technology II Catholic University of America Washington DC USA; ^11^ Mullard Space Science Laboratory University College London Surrey UK; ^12^ Department of Mathematics, Physics and Electrical Engineering Northumbria University Newcastle Upon Tyne UK; ^13^ Space Research Institute Austrian Academy of Sciences Graz Austria; ^14^ Department of Astrophysical and Planetary Sciences University of Colorado Boulder Boulder CO USA

**Keywords:** energetic electrons, flux rope, reconnection X‐line, adiabatic energy, Betatron and Fermi acceleration

## Abstract

The properties and acceleration mechanisms of electrons (<200 keV) associated with a pair of tailward traveling flux ropes and accompanied reconnection X‐lines in Earth's plasma sheet are investigated with MMS measurements. Energetic electrons are enhanced on both boundaries and core of the flux ropes. The power‐law spectra of energetic electrons near the X‐lines and in flux ropes are harder than those on flux rope boundaries. Theoretical calculations show that the highest energy of adiabatic electrons is a few keV around the X‐lines, tens of keV immediately downstream of the X‐lines, hundreds of keV on the flux rope boundaries, and a few MeV in the flux rope cores. The X‐lines cause strong energy dissipation, which may generate the energetic electron beams around them. The enhanced electron parallel temperature can be caused by the curvature‐driven Fermi acceleration and the parallel electric potential. Betatron acceleration due to the magnetic field compression is strong on flux rope boundaries, which enhances energetic electrons in the perpendicular direction. Electrons can be trapped between the flux rope pair due to mirror force and parallel electric potential. Electrostatic structures in the flux rope cores correspond to potential drops up to half of the electron temperature. The energetic electrons and the electron distribution functions in the flux rope cores are suggested to be transported from other dawn‐dusk directions, which is a 3‐dimensional effect. The acceleration and deceleration of the Betatron and Fermi processes appear alternately indicating that the magnetic field and plasma are turbulent around the flux ropes.

## Key Points

- Fermi and parallel potential is strong near X‐lines, Betatron is strong on flux rope boundaries, and electrostatic structures in flux rope
- Adiabatic electrons are to a few keV around X‐line, 10's keV downstream of X‐line, 100's keV on flux rope boundary and a few MeV in flux rope
- Energetic electrons and electron distribution function suggest that field lines in flux rope core connect to X‐line in dawn‐dusk direction

## 1 Introduction

The acceleration mechanism for energetic particles is one of the fundamental science questions in space and astrophysical plasma physics. Magnetic reconnection, which can convert magnetic energy into plasma kinetic and thermal energies, is believed an effective way of energizing particles (Yamada et al., 2010; Zweibel & Yamada, 2009). Meanwhile, magnetic reconnection often produces coherent magnetic structures, for example, flux ropes or plasmoids in spacecraft observations and magnetic islands in 2‐dimensional (2D) simulations, which are proposed to play important roles in accelerating particles (Drake, Swisdak, Che, & Shay, 2006). Recent studies have shown that strong turbulence associated with magnetic reconnection in the Earth's magnetotail can significantly accelerate particles, including the relativistic electrons with energies higher than 10s keV (Ergun et al., 2020a, 2020b).

In the reconnection diffusion region, where magnetic field lines reconnect, particles are accelerated by the non‐ideal electric field as shown in theoretical and simulations studies, see for example, Hoshino et al. (2001), X. R. Fu et al. (2006), and Egedal et al. (2012). Electrons can be accelerated by the parallel electric field near the separatrices, that is, trapped electron model, where electrons are trapped by the mirror force or the parallel potential drops (Egedal et al., 2012). Electrons may also be accelerated by the structures that carry the parallel electric field, such as electrostatic solitary waves (ESWs) (Drake et al., 2003), double layers (Egedal et al., 2015), and slow shocks in the low plasma β reconnection (Q. Zhang et al., 2019a, 2019b). Electron acceleration in the exhaust jets immediately downstream of active reconnection sites but within the ion diffusion region might result from electron re‐magnetization (Arnold et al., 2021; Drake et al., 2009; Li et al., 2017). Following the acceleration in and around the immediate vicinity of a reconnection X‐line, particles are suggested to be further accelerated by Betatron and/or Fermi‐type acceleration processes downstream of the reconnection site (Hoshino et al., 2001; Pellinen & Heikkila, 1978; Scholer, 1984; S. Wang et al., 2016).

In the studies related to magnetic islands, it has been suggested that the contraction of magnetic islands can result in Fermi‐reflection electron accelerations (Drake, Swisdak, Che, & Shay, 2006). The Fermi‐reflection acceleration refers to electrons trapped in closed magnetic field lines, and they would continuously gain energy when passing through the flows driven by contracting magnetic field lines repeatedly (Drake, Swisdak, Che, & Shay, 2006; Li et al., 2021). Electrons gain significant energy through the curvature‐driven Fermi bounce mechanism and re‐magnetizations by the flows through each crossing (Li et al., 2021). Coalescence of neighboring magnetic islands through a secondary reconnection can repeatedly accelerate the trapped electrons through reconnection electric fields and further Fermi‐reflection acceleration (Arnold et al., 2021; Hoshino, 2012; Oka et al., 2010; Pritchett, 2008). In some 2D simulations, the electrons can be confined within the magnetic island and accelerated to high energy (Lu et al., 2020). In 3‐dimensional (3D) fully kinetic simulations, Q. Zhang et al. (2021) show that the flux rope kink instability results in strong field line chaos during the magnetic reconnection with a weak guide field. The field line chaos allows particles to transport out of the flux ropes and then further be accelerated by the reconnection electric field.

In the Earth's magnetotail, magnetic flux ropes or plasmoids have been observed with scales from several *R*_*E*_ (*R*_*E*_ is one Earth radius) to sub‐ion scales (smaller than ion inertial length) (see e.g., L. J. Chen et al., 2007; Hones, 1984; Ieda et al., 1998; Slavin et al., 1989, 2003; Sun et al., 2019). Energetic particles, that is, both ions and electrons, are observed to be accompanied by large‐scale (several *R*_*E*_) flux ropes, or plasmoids (e.g., Hones, 1984; Scholer et al., 1985; Slavin et al., 1990; Zong et al., 1997, 2004), and the ion‐scale flux ropes (e.g., L. J. Chen et al., 2007; Huang et al., 2012; Retinò et al., 2008; R. Wang et al., 2010; Zhong et al., 2020). Energetic electrons have been also detected in the reconnection diffusion regions (e.g., G. Chen et al., 2019; Cohen et al., 2021; H. S. Fu et al., 2019; Huang et al., 2012; Imada et al., 2007; Øieroset et al., 2002; Oka et al., 2016; Turner et al., 2021; R. Wang et al., 2010; Zong & Zhang, 2018) and in the reconnection outflow pileup regions (e.g., H. S. Fu et al., 2013; Imada et al., 2007; Nakamura et al., 2013; Xu et al., 2018). The enhancements of energetic electrons are observed in the energy up to a few hundred keV in most of the above studies. Some studies report the enhancements of a few MeV electrons in the deep magnetotail (i.e., downtail ∼100 *R*_*E*_), which are shown to be accompanied by substorms (Krimigis & Sarris, 1980; Richardson et al., 1996; Slavin et al., 1992).

The consequence of net acceleration by these different mechanisms is that particles typically develop power‐law energy spectra. In the Earth's magnetotail, many measurements have shown the power‐law spectra of energetic electrons, and the slopes of the spectra vary in different structures or regions. For example, a diversity of power‐law spectra are found for ions and electrons in the plasma sheet (see Christon et al., 1991; Espinoza et al., 2018; Sun et al., 2018), and reconnection diffusion region (see Cohen et al., 2021; Huang et al., 2012; Øieroset et al., 2002; Oka et al., 2016).

Measurements from the Magnetospheric MultiScale (MMS) mission (Burch, Moore, et al., 2016) have directly revealed the process of energy converted from electro‐magnetic energy into particle thermal and kinetic energy in and around the reconnection electron diffusion region (EDR) (Burch, Torbert, et al., 2016). Measurements from MMS have also enabled investigations of intensities of the local Betatron, local curvature‐driven Fermi, parallel electric field, and non‐adiabatic/nonlinear acceleration processes. For example, the local Betatron, local curvature‐driven Fermi and local parallel electric field associated with flux transfer events at the dayside magnetopause (Akhavan‐Tafti et al., 2019), reconnection outflow (Eriksson et al., 2020) and flux ropes in Earth's cross‐tail current sheet (Jiang et al., 2021; Zhong et al., 2020) have been investigated.

The study of energetic particles associated with flux ropes and X‐lines based on in situ measurement is of significant interest. The in situ measurements can reveal the distributions of energetic electrons, and also the roles of the various acceleration mechanisms. MMS has provided unprecedented high spatial and temporal measurements of fields and particles, which enable comprehensive investigations of the properties of electrons and acceleration mechanisms. Moreover, previous studies based on in situ measurements frequently investigate the energetic particles associated with flux ropes and X‐lines, separately. In this study, based on the MMS measurements, we analyze the properties of electrons associated with a pair of flux ropes and X‐lines near them. We discuss the acceleration mechanisms associated with the flux rope pair and the reconnection X‐lines.

Our paper is organized as follows. Section 2 provides an overview of the MMS measurements across a pair of flux ropes bounded by X‐lines. The general profile of the flux ropes is determined using the Grad‐Shafranov (GS) reconstruction technique, and the features of the X‐lines are shown. In Section 3, the properties of the energetic electrons, including the variations in particle fluxes, the pitch angle distributions, and the power‐law spectra of the energetic electrons (>50 keV), are shown. In Section 4, the energies of the adiabatic electrons are analyzed based on the ratio of the local magnetic field curvature radius to the gyro‐radius of the electrons. The energization of electrons due to the local Betatron, local curvature‐driven Fermi, and the local parallel electric field processes are analyzed. Electron distribution functions with energy lower than 30 keV and the accelerations due to parallel electric potentials are discussed. Section 5 discusses the energization mechanisms associated with the flux rope pair and X‐lines, the power‐law spectra of electrons, including the discussion of Fermi‐reflection, and the 3‐dimensional effect. Section 6 is devoted to the conclusions of this study.

## 2 Flux Rope Pair and Reconnection X‐Lines on 12 July 2017

### 2.1 MMS Data and Instrument

This study utilizes field and particle measurements from the MMS mission (Burch, Moore, et al., 2016). The magnetic field measurements are from the fluxgate magnetometers (FGM) (Russell et al., 2016). In burst mode, the FGM provides the measurement of magnetic field vectors at a sampling rate of 128 vectors/s. The electric field measurements are from the combination of the axial double probe (ADP) (Ergun et al., 2016) and the spin plan double probes (SDP) (Torbert et al., 2016). The combination provides the measurements of the DC electric field vectors.

The low‐energy electrons and ions are from the measurements of the fast plasma investigation (FPI) (Pollock et al., 2016). The FPI, in burst mode, measures 3D distributions of electrons and ions with the energy range of ∼0.01–30 keV/*q* at time resolutions of 30 and 150 ms, respectively. We also employ the proton measurements from the Hot Plasma Composition Analyzers (HPCA) (D. T. Young et al., 2016). The HPCA can measure four ion species (H^+^, He^++^, He^+^, and O^+^) with energies from ∼1 eV to 40 keV/*q* at a time resolution of 10 s. The energetic electrons are measured by the Fly's Eye Electron Proton Spectrometer (FEEPS) (Blake et al., 2016). FEEPS provides full 3D distributions of energetic (∼47.2–∼550 keV) electron differential energy intensities at a time resolution of 0.3125 s in burst mode.

### 2.2 Overview of MMS Measurements

**Figure 1:**
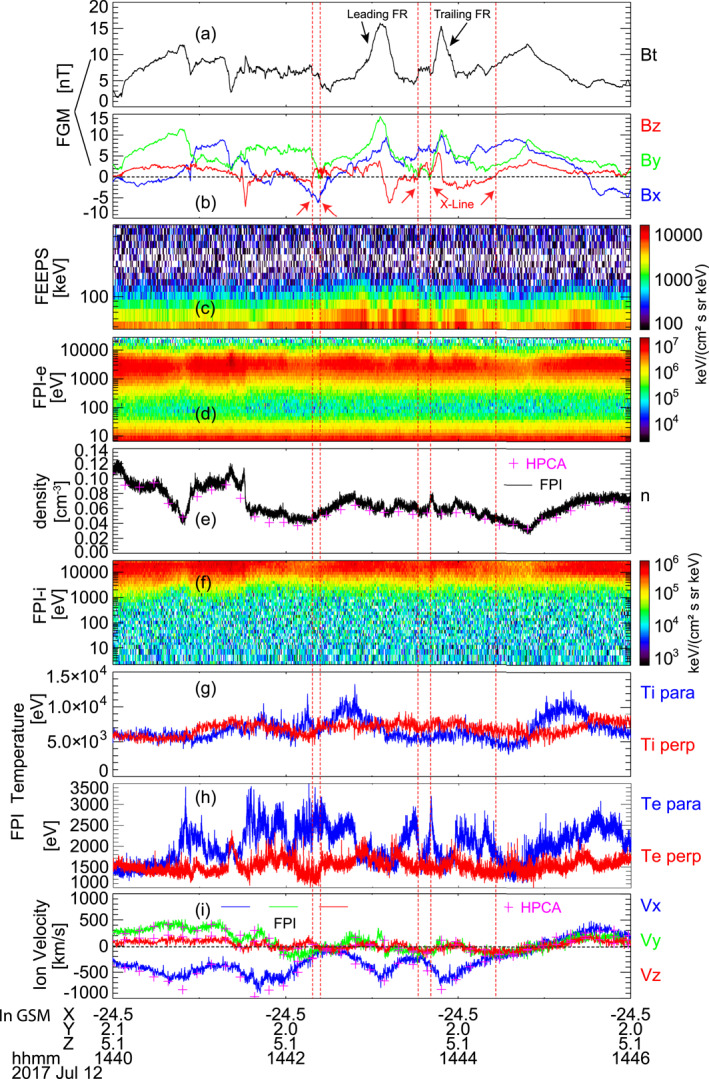
Overview of the measurements from MMS1 on 12 July 2017 between 14:40 to 14:46 UTC. (a) Magnetic field intensity (*B*_*t*_). (b) Magnetic field components, *B*_*x*_ in blue, *B*_*y*_ in green, *B*_*z*_ in red. (c) Energetic energy spectrum from FEEPS (>47.2 keV). (d) Electron energy spectrum from FPI (<30 keV). (e) Electron number density measured by FPI (solid line) and ion number density measured by HPCA (crosses). (f) Ion energy spectrum from FPI (<30 keV). (g) Ion and (h) electron temperature, the perpendicular component is marked in red and the parallel component in blue. (i) Ion bulk velocity measured by FPI and HPCA, the solid lines are from FPI, and the crosses are from HPCA. In the electron momentum, low‐energy photo‐electrons are removed (Gershman et al., 2017). In panel (a), the flux rope observed first is termed “Leading FR,” and the flux rope following it is termed “Trailing FR.” Both flux ropes correspond to bipolar *B*_*z*_ and strong fields in the centers (*B*_*y*_ and *B*_*t*_). The vertical dashed red lines indicate the reconnection X‐lines surrounding the flux ropes. The measurements in panel (b) and (i) are shown in the Geocentric Solar Magnetospheric (GSM) coordinate system.

On 12 July 2017 from 14:40 to 14:46 coordinated universal time (UTC), MMS was located ∼24.5 *R*_*E*_ (*R*_*E*_, one Earth radius, 6,371 km) downtail from Earth in the plasma sheet. It encountered strong tailward plasma flows accompanied by a pair of tailward traveling flux ropes. Figure 1 shows the magnetic field and particle measurements from MMS during this period. The two flux ropes were accompanied by *B*_*z*_ bipolar signatures from positive to negative and enhancements in *B*_*y*_ and *B*_*t*_ (Figures 1a and 1b). The leading flux rope (leading FR) was centered at ∼14:43:05 UTC, and the trailing flux rope (trailing FR) was centered at ∼14:43:50 UTC. As shown in Figure 1c, the energy fluxes of energetic electrons with energies between ∼47.2 and 200 keV were significantly enhanced around the flux rope pair compared with the prior reconnection outflow (14:40 to 14:42 UTC). The electron temperature in Figure 1h shows that the parallel temperature of electrons exhibits several peaks with intensities higher than the perpendicular temperature.

MMS crossed an active plasma sheet before the flux rope pair. Figure S1 in Supporting Information S1 includes the measurements from 14:00 to 15:00 UTC. The nearest possible quiet plasma sheet crossing made by MMS was between 14:12 to 14:19 UTC. In comparison with the quiet plasma sheet, the enhancements of the energetic electrons of the flux rope pair were strong in both higher energy and stronger fluxes.

The speed of the tailward flow reached small values during three short periods in Figure 1i, which were at ∼14:42:30 UTC (before the leading flux rope), 14:43:30 UTC (between the two flux ropes), and 14:44:55 UTC (after the trailing flux rope). The tailward flow converted into earthward after ∼14:44:55 UTC and the *B*_*z*_ changed from negative to positive ∼30 s earlier indicating an X‐line crossing.

### 2.3 Grad‐Shafranov Reconstruction of Flux Rope Pair

**Figure 2:**
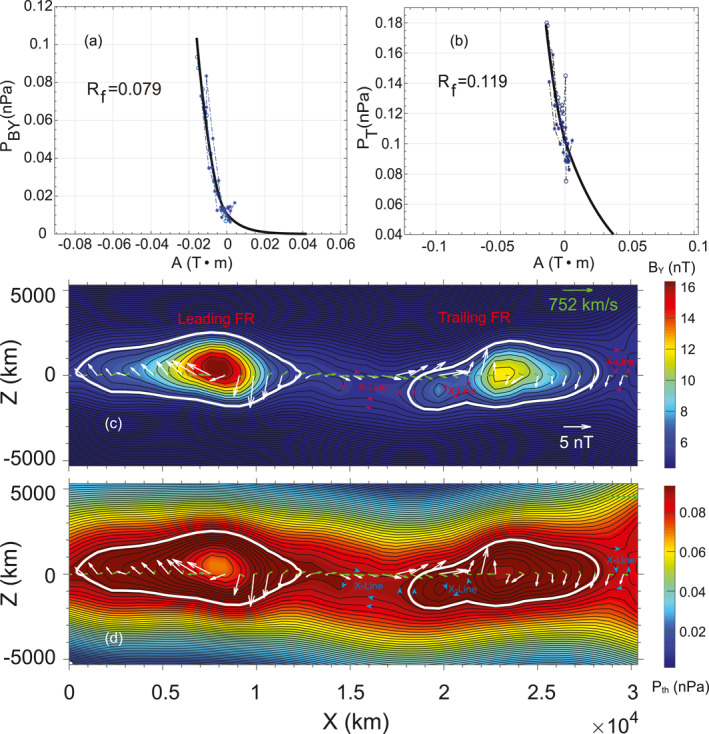
Grad‐Shafranov (G‐S) reconstruction of the flux rope pair between 14:42:45 and 14:44:15 on 20 July 2017. (a and b) The magnetic pressure of the core field $\left(P_{BY}=B_{Y}^{2}/2\mu_{0}\right)$ and the transverse pressure $\left(P_{T}=p_{th}+B_{Y}^{2}/2\mu_{0}\right)$ versus magnetic potential vector A. The black lines are the polynomial fits of the circles. The circles are the measurements from MMS1. R_f_ represents the fitting residue. (c and d) The reconstructed cross‐section maps of the axial field intensity (*B*_*Y*_) and the thermal pressure (*p*_*th*_). The white and green arrows indicate the measured *B*_*XZ*_ and remaining ion flow in the frame of flux ropes. The white contours represent the boundaries of the flux ropes. The Leading FR, Trailing FR, and “X‐lines” are labeled in (c) and (d). The “X‐line” are possible X‐lines. Note that the last three X‐lines in Figure 1 correspond to the three possible X‐lines revealed by G‐S reconstruction.

The Grad‐Shafranov (GS) reconstruction technique (Hau & Sonnerup, 1999; Hu & Sonnerup, 2002, 2003; Sonnerup & Guo, 1996) was applied to investigate the structures of the flux rope pair between 14:42:45 and 14:44:10 UTC. Figure 2 shows the reconstructed maps of the core magnetic field (*B*_*Y*_) and the thermal pressure (*p*_*th*_) of the flux rope pair. The GS reconstruction required a few assumptions when applying it to the magnetic structures. First, the magnetic structure shall be time‐independent in a proper frame of reference. Second, the gradients in the axial direction (defined by $\vec{Y}$) are much smaller than those in the reconstructed plane (defined by $\vec{X}$ and $\vec{Z}$). Third, the structure can be deemed as quasi‐magnetohydrostatic when the spacecraft crosses it (∂/∂t∼0). As a result, the Hall term solely balances the gradient of the thermal pressure

(1) $$\nabla p_{th}=\vec{J}\times \vec{B},$$

where *p*_*th*_ is the thermal pressure, $\vec{J}$ and $\vec{B}$ are the current density and magnetic field vectors. In this equation, the particle distribution is assumed to be isotropic and *p*_*th*_ is a scalar. Considering Ampere's law $\left(\nabla \times \vec{B}=\mu_{0}\vec{J}\right)$ and using magnetic vector potential ($\vec{A}$) to describe the transverse magnetic field $\vec{B}=\left[\partial A/\partial Z,-\partial A/\partial X,B_{Y}\left(A\right)\right]$, the Grad‐Shafranov equation (Sturrock, 1994) can be derived as

(2) $$\partial^{2}A/\partial X^{2}+\partial^{2}A/\partial Z^{2}=-\frac{\mu_{0}d\left(p_{th}+\frac{B_{Y}^{2}}{2\mu_{0}}\right)}{dA}.$$

In the GS equation, the pressure in the transverse plane $\left(P_{T}=p_{th}+B_{Y}^{2}/2\mu_{0}\right)$ is a function of the A. During the reconstructing interval between 14:42:45 and 14:44:10 UTC including the flux rope pair, the ion temperature anisotropy was weak (Figure 1g) with the perpendicular temperature larger than the parallel temperature. As demonstrated by Hasegawa et al. (2005), weak temperature anisotropy should have limited effects on the reconstruction.

Building the appropriate local coordinate and finding the frame of reference are keys to the GS reconstruction technique. We first employed the Minimum Directional Derivative or Difference (MDD) method (Shi et al., 2005, 2019) to find the axial direction for the flux ropes ($\vec{Y}$). Then we employed the axial searching method developed by Hu and Sonnerup (2002) to obtain an improved axial direction. This axial searching method is to minimize the differences between the *P*_*T*_, which were obtained by integrating the inbound and outbound paths crossing the flux ropes from the MMS in situ measurements. The P_T_ is known as a double folding pattern for a flux rope crossing. The ${\vec{Y}}_{MDD}$ were (0.321, 0.931, 0.171) and (0.313, 0.949, 0.051) for the leading FR and trailing FR, which were only separated by ∼7°. The close separation of the axial directions between the two flux ropes enabled us to reconstruct them together. The axial finding technique was based on the ${\vec{Y}}_{MDD}$ and determined the $\vec{Y}$ = (−0.149, 0.962, 0.231), which suggested that the flux rope axes were both predominantly located in the dawn‐dusk direction. The MDD verified that the magnetic gradient along the ${\vec{Y}}_{\mathrm{MDD}}$ was indeed much smaller than the gradients along other two directions. The frame of reference of the flux rope pair was determined from the de Hoffmann‐Teller analysis (HT) (Khrabrov & Sonnerup, 1998), which was (−550, 70, −40) km/s (see Appendix A), corroborating the strong anti‐planetward flow noted above. Appendix A also shows the result of Walén test.

The magnetic field vector potential A was calculated by integrating the *B*_*Y*_ along the trajectory of the spacecraft. The magnetic vector potential A at *Z* distance of the spacecraft trajectory was calculated through the Taylor expansion (Hau & Sonnerup, 1999; Sonnerup & Guo, 1996). The values of the magnetic vector potential A near the boundaries of the reconstructed area were determined by the technique introduced by Hu and Sonnerup (2003).

Figures 2a and 2b show the polynomial fittings of the magnetic pressure of the core field $\left(P_{BY}=B_{Y}^{2}/2\mu_{0}\right)$ and the transverse pressure $\left(P_{T}\right)$ to the magnetic vector potential A, respectively. Based on the fitting relations, the core field and the transverse pressure were derived from the reconstructed map of the A. Figures 2c and 2d demonstrate the intensities of *B*_*Y*_ and *p*_*th*_. The intensities of the *p*_*th*_ were obtained by *P*_*T*_ minus *P*_BY_. The core fields were significantly enhanced in the centers of flux ropes and the thermal pressure was depleted in the centers compared with the boundaries.

The two white contours represented the boundaries of the flux ropes. The leading flux rope had a scale of ∼15,000 km along the X‐direction, which corresponded to ∼16 ion inertial lengths (*d*_*i*_). In this study, FPI measurements of the electron density were used as the plasma density, which was reasonably consistent with the proton density measured by HPCA (Figure 1e). The plasma density was ∼0.06 cm^−3^ on average, which corresponded to an ion inertial length (d_i_) of ∼930 km. The scale along the *Z* direction for the leading flux rope was ∼5,000 km, which corresponded to ∼5.3 *d*_*i*_. The trailing flux rope had a scale of ∼10,000 km along the *X* direction, which was ∼10.5 *d*_*i*_. The scale along the *Z* direction was ∼4,000 km, which was ∼4.2 *d*_*i*_. The scale was smaller for the tailing flux rope than the leading flux rope. The strengths of the *B*_*Y*_ and the *p*_*th*_ were weaker of the trailing flux rope than the leading flux rope. In the reconstructed map, two possible reconnection X‐lines appeared between the flux rope pair, which were marked in Figure 2c.

### 2.4 Reconnection X‐Lines Around Flux Rope Pair

**Figure 3:**
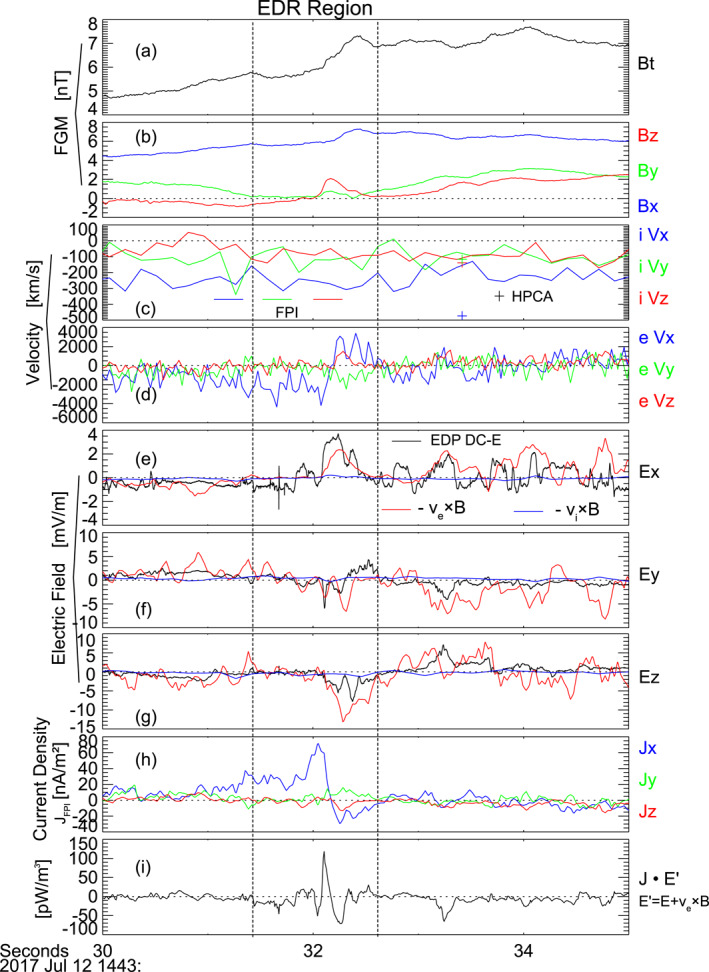
Observations of an active X‐line between the two flux ropes. (a) Magnetic field intensity (*B*_*t*_). (b) Magnetic field components, *B*_*x*_ in blue, *B*_*y*_ in green, *B*_*z*_ in red. (c) Ion bulk velocity measured by FPI and HPCA, the solid lines are from FPI, and the crosses are from HPCA. (d) Electron bulk velocity from FPI. (e, f, and g) Electric field x component, y component, and z component. Black lines are the measurements of EDP DC electric fields, blue lines are ion convection electric fields $\left(-{\vec{v}}_{i}\times \vec{B}\right)$, red lines are electron convection electric field $\left(-{\vec{v}}_{e}\times \vec{B}\right)$. (h) Current density calculated from particle measurements, $n_{e}e\left({\vec{v}}_{i}-{\vec{v}}_{e}\right)$. (i) Energy dissipation rate $\vec{J}\cdot {\vec{E}}^{\prime }$, where ${\vec{E}}^{\prime }=\vec{E}+{\vec{v}}_{e}\times \vec{B}$.

Figure 3 shows the observations of an X‐line region between the two flux ropes at ∼14:43:32 UTC. In Figure 3b, *B*_*z*_ changed from negative to positive which was accompanied by the reversal of electron flow direction from tailward to earthward (Figure 3d). The speeds of the *x* component of electron bulk velocity (*v*_*x*_) changed from ∼−4,000 to 3,000 km/s. Both the magnetic field and electron velocity changing directions indicated that an X‐line region passed the spacecraft. The electron flow reversed in the *x* direction and the magnetic field reversed in the *z* direction suggested that the reconnection X‐line reconnected the *x* component of the magnetic field, that is, the reconnection was separating the flux rope pair.

Figures 3e–3g compare the DC electric field and the convection electric fields of ions and electrons. The differences between the DC electric field (black lines) and the convection electric field of ions (blue lines) indicated that ions were not frozen‐in. Meanwhile, there were large differences between the DC electric field and the convection electric field of electrons (red lines).

On the other hand, the current density was strong with peak intensities of ∼75 nA/m^2^ (Figure 3h), and the energy dissipation rate $\left(\vec{J}\cdot {\vec{E}}^{\prime }\right)$, where ${\vec{E}}^{\prime }$ was the electric field in the frame of convection electrons $\left(\vec{E}+{\vec{v}}_{e}\times \vec{B}\right)$, was strong as well. The values of $\vec{J}\cdot {\vec{E}}^{\prime }$ were both negative and positive with the largest positive value of ∼110 pW/m^3^. Meanwhile, the electron v_x_ reversed from tailward into earthward. All suggested that MMS crossed an electron diffusion region (EDR). The EDR is accompanied by strong energy dissipation and significantly enhanced out‐of‐plane electron bulk flow, that is, the *y* direction in our case, as shown in both simulations and observations (e.g., Burch, Torbert, et al., 2016; Phan et al., 2018; Zenitani et al., 2011). However, MMS was not close enough to the current sheet center in this EDR event since the *B*_*x*_ remained large and the electron *v*_*y*_ was not significantly enhanced. The negative values of the $\vec{J}\cdot {\vec{E}}^{\prime }$ were often observed in the EDRs, especially in the outer EDRs (see Daughton et al., 2006; Hwang et al., 2017; Karimabadi et al., 2007; Xiong et al., 2022). In our event, since ion bulk velocities were tailward with *v*_*x*_ of ∼−300 km/s (Figure 3c) during the entire period in Figure 3, it was likely that ions did not participate in this reconnection X‐line and this reconnection X‐line was an electron‐only reconnection (see Phan et al. [2018] and Zhou et al. [2021] for examples of electron‐only reconnection).

There were other reconnection X‐lines around the flux rope pair, which are listed in Table 1. They were located at ∼14:42:18, 14:42:23, 14:43:40, and 14:44:30 UTC. The first three reconnection X‐lines corresponded to electron flow reversals, magnetic field direction changes, strong electric field and current density, and strong energy dissipation $\left(\vec{J}\cdot {\vec{E}}^{\prime }\neq 0\right)$. They did not correspond to clear direction changes in ion velocity around the time when electron flow reversals. Therefore, the first three reconnections were likely electron‐only reconnections similar to the one shown in Figure 3. The last X‐line corresponded to *B*_*z*_ reversal at ∼14:44:25 UTC and *V*_*i*_ reversal at ∼14:44:52 UTC, which was likely a reconnection X‐line involving ions. However, since MMS was located further away from the neutral sheet (*B*_*x*_ > 5 nT) during this event, it did not encounter the EDR. The first and second reconnection X‐lines correspond to strong negative values of $\vec{J}\cdot {\vec{E}}^{\prime }$, which may indicate that the MMS encounters the outer EDRs.

**Table 1:** Reconnection X‐Lines Around the Flux Rope Pair

Time (UTC)	Peak intensity of current density (nA/m^2^)	Peak intensity of $\vec{J}\cdot {\vec{E}}^{\prime }$ (pW/m^3^)
14:42:18	50	−85
14:42:23	33	−112
14:43:32 (Figure 3)	75	110
14:43:40	39	82
14:44:25	15	25

Therefore, around the flux rope pair, five reconnection X‐lines were identified. Two of the reconnection X‐lines were located between the flux rope pair. Four of the five reconnection X‐lines were likely electron‐only reconnection. We note that it does not mean that there are only these five reconnection X‐lines near the flux rope pair. Our selection criteria require strong electric fields, current densities, and energy dissipation. The spatial scale of the distances between MMS's four spacecraft was in the scale of electron inertial length. Therefore, there may be reconnection X‐lines that are not captured by the MMS. The properties of electron‐only reconnection X‐lines around the flux ropes are consistent with the recent observations of electron‐only reconnections in the turbulent magnetosheath (Phan et al., 2018), turbulent foreshock region (Liu et al., 2020) and turbulent plasma sheet (see Zhou et al., 2021).

## 3 Distributions of Energetic Electrons

### 3.1 Energetic Electrons Associated With Flux Rope Pair and X‐Lines

**Figure 4:**
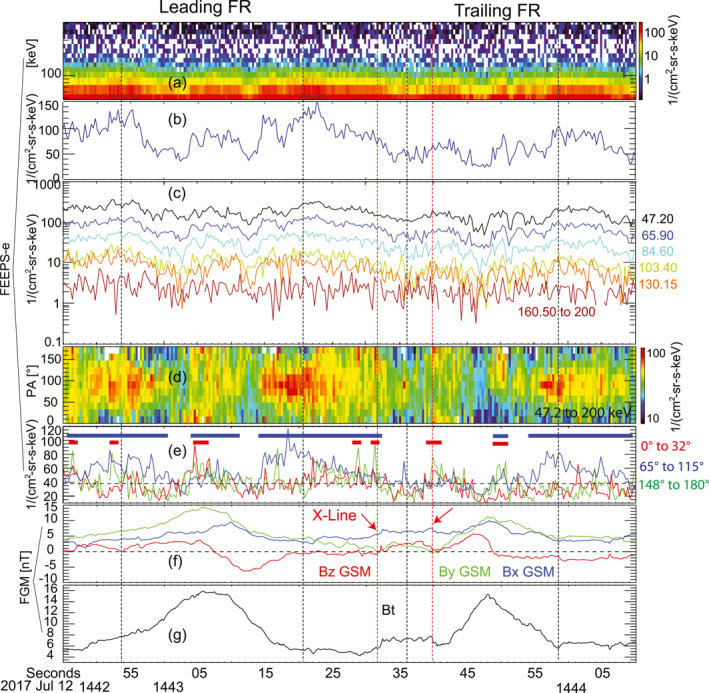
Observations of the energetic electrons from FEEPS associated with the flux rope pair and the X‐lines. (a) Energetic‐electron energy spectrum (>47.2 keV). (b) Differential particle fluxes of electrons within the energy channel centered at 65.9 keV. (c) Differential particle fluxes of energetic electrons within energy channels up to 200 keV. (d) Pitch angle distribution of energetic electrons with energy from ∼47.2 to 200 keV. (e) Average particle fluxes of energetic electrons of pitch angle bins in the parallel direction including pitch angle bins of 0°–16.35°, 16.35°–32.7° (the red line), perpendicular direction including pitch angle bins of 65.46°–81.82°, 81.82°–98.18°, 98.18°–114.54° (the blue line), and antiparallel direction including pitch angle bins of 147.27°–163.63°, 163.63°–180° (the green line). The horizontal dashed line indicates the mean particle flux of the pitch angle bins during the period of this figure, which is ∼38.4 (cm^2^ sr s keV)^−1^. The blue bars indicate the periods corresponding to the enhancements of perpendicular electrons, and the red bars indicate the periods of enhancements of parallel electrons. (f) Magnetic field components, *B*_*x*_ in blue, *B*_*y*_ in green, *B*_*z*_ in red. (g) Magnetic field intensity, *B*_*t*_. The dashed red vertical lines indicate the possible reconnection X‐lines.

Figure 4 shows the properties of energetic electrons measured by FEEPS between 14:42:45 and 14:44:10 UTC, which includes the flux rope pair and the X‐lines in between. Both the leading and trailing flux ropes corresponded to energetic electron enhancements on the outer boundaries, including the front and rear boundaries, and the core. The particle fluxes within energy channels from 47.2 to 200 keV in Figures 4b and 4c are displayed as the “*W*” profile. The variations of electron fluxes did not show clear energy dependencies, that is, the increase or decrease of electron fluxes were similar within different energy channels (Figure 4c). Only the forward boundary of the trailing flux rope does not correspond to an obvious increase of electron fluxes in the energy range from 160.5 to 200 keV.

Figures 4d and 4e show the pitch angle information of the energetic electrons associated with the flux rope pair and reconnection X‐lines. It demonstrated that the energetic electron was highest in the direction perpendicular to the local magnetic field. The enhancements of energetic electrons in the perpendicular direction appeared particularly on outer boundaries and also in the core of both flux ropes. There were also enhancements of energetic electrons in parallel or antiparallel directions during this period. Different from the enhancements in the perpendicular direction persisting ∼10 s, the enhancements in the parallel or antiparallel directions are exhibited as beams. These beams often persisted in a short time scale of a second or a few seconds, and they often appeared unidirectional. A unidirectional beam appeared in the cores of the trailing flux rope at ∼14:42:50 UTC. The reconnection X‐lines between the two flux ropes corresponded to clear energetic electron beams (Figure 4e) in the antiparallel direction at ∼14:43:31 UTC and bidirectional beams at ∼14:43:40 UTC.

### 3.2 Power Law Spectra

**Figure 5:**
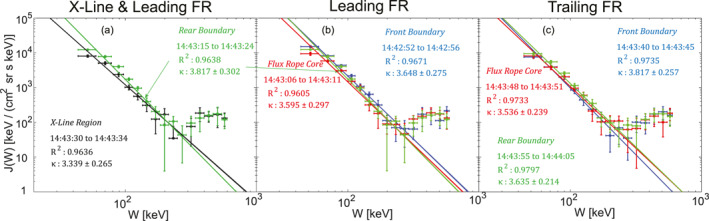
The power law fittings $\left(J\left(W\right)\propto W^{-\kappa }\right)$ of the differential energy flux *J*(*W*) in the flux ropes and around the reconnection X‐lines. Panel (a) black dots with errorbars are the measurements around the X‐line region (from 14:43:30 to 14:43:34 UTC), the black line represents the fitting result of the power‐law distribution. Green dots and the green line are for the measurements and fitting on the rear boundary of the leading flux rope. (b) The colors in blue, red, and green represent the measurements and fittings on the front boundary, the core, and the rear boundary of the leading flux rope centered at ∼14:43:08 UTC. Panel (c) is in the similar format as (b) and is for the trailing flux rope centered at ∼14:43:50 UTC. The values of the *R* ^2^ represent the square of the correlation coefficient obtained by the linear regression fitting of log_10_(*J*(*W*)) and log_10_(*W*). The power‐law fitting includes the data points with energies between ∼47.2 and ∼200 keV, including the measurements from eight energy channels. The horizontal errorbars correspond to the widths of the energy channels and the vertical errorbars correspond to the standard deviations of the measurements within each time interval. The bumps on the tails of the *J*(*W*) with energies higher than 300 keV are due to cosmic rays (See Cohen et al. [2021] on the contaminations by cosmic rays).

The differential energy flux *J*(*W*) from the FEEPS was fitted by the power‐law distributions $\left(J\left(W\right)\propto W^{-\kappa }\right)$. Figure 5a shows the distributions of *J*(*W*) versus W around the reconnection X‐line and on the rear boundary of the leading flux rope. The spectrum of the energetic electrons near the reconnection X‐line was averaged from 14:43:30 to 14:43:34 UTC, which corresponded to a κ value of ∼3.34. The other reconnection X‐line between the flux rope pair was observed at ∼14:43:40 UTC, whose energetic electron spectrum corresponded to κ value of ∼3.63. The spectrum on the rear boundary was averaged from 14:43:15 to 14:43:24 UTC corresponding to a κ value of ∼3.82. Thus, the energy spectrum was slightly harder near the reconnection X‐lines than on the boundary of the leading flux rope. The κ differences between the spectra near the X‐lines and the rear boundary of the leading flux rope were ∼14% and 5%, which were comparable to the 8% uncertainties of the fittings resulting from the linear regressions.

Figures 5b and 5c show the spectra and power‐law fittings of the *J*(*W*) of the outer boundaries and core of the flux rope pair. For the leading flux rope, the fluxes of the energetic electrons in the core were lower compared to those on the front and rear boundaries. The κ in the flux rope core (3.60) was slightly harder than the κ on the boundaries (3.65 and 3.82). However, the differences in the κ were small <10%, which was comparable to the uncertainties resulting from the fittings. Therefore, the decrease of the κ in the flux rope core was not significant compared to those on the boundaries. For the trailing flux rope, the κ of the spectra show a similar feature to the leading flux rope. The κ in the flux rope core (∼3.54) was slightly smaller than those κ at the front and rear boundaries (∼3.82 and ∼3.64), but the decreases were not significant compared to the uncertainties.

**Figure 6:**
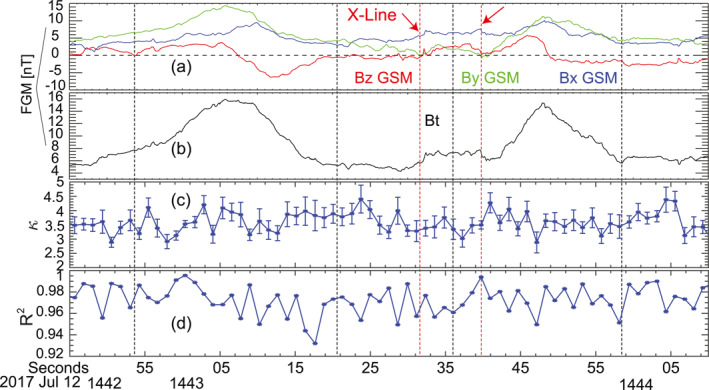
The distribution of the κ values of the energetic electrons measured by MMS1 FEEPS. (a) Magnetic field components, *B*_*x*_ in blue, *B*_*y*_ in green, *B*_*z*_ in red. (b) Magnetic field intensity, *B*_*t*_. (c) The values of κ obtained from the power‐law fitting. (d) The square of the correlation coefficient (*R* ^2^) obtained by the linear regression fitting of log_10_(J(W)) and log_10_(W). In (c and d), one fitting point corresponds to the average of four data points.

To summarize the variations of the spectra slope, Figure 6c shows the values of the κ in time sequences. Figure 6d shows the square of the correlation coefficient (*R* ^2^) of the power‐law fitting. Each fitting corresponds to the average of four data points measured by FEEPS. The values of *R* ^2^ are higher than 0.95 for most points indicating that the distributions are well‐fitted. It can be seen that the κ values are indeed smaller near the reconnection X‐lines (the two dashed vertical lines) compared with surrounding regions.

In conclusion, the electron spectra near the reconnection X‐lines were harder than the electron spectra on the boundaries of the flux ropes. The electron spectra in the flux rope cores were slightly harder than those spectra on the boundaries, but likely within the uncertainties.

## 4 Energization of Electrons Associated With Flux Ropes and X‐Lines

### 4.1 Energization Under Guiding Center Approximation

The derivation of the total kinetic energy density (*dU*/*dt*) of electrons can be written under the guiding center approximation (Dahlin et al., 2014; Northrop, 1963)

(3) $$\frac{dU}{dt}=E_{\|}J_{\|}+\frac{p_{⊥}}{B}\left(\frac{\partial B}{\partial t}+{\vec{v}}_{E}\cdot \nabla B\right)+\left(p_{\|}+m_{e}n_{e}v_{\|}^{2}\right){\vec{v}}_{E}\cdot \left(\vec{b}\cdot \nabla \vec{b}\right),$$

where $E_{\|}$ and $J_{\|}$ are the parallel electric field and current density, $p_{⊥}$ and $p_{\|}$ are the perpendicular and parallel thermal pressure, ${\vec{v}}_{E}=\vec{E}\times \vec{B}/B^{2}$ corresponds to the drifting velocity due to the convection electric field, $\vec{b}$ is the magnetic field vector. It is noted that $p_{⊥}$, $p_{\|}$, $n_{e}$, and $v_{\|}$ in the equation are the integral of electrons as long as they are quasi‐adiabatic and the guiding center approximation is valid. Thus, this equation represents the bulk acceleration/heating of electrons.

On the left‐hand side, the total kinetic energy contributes by two terms. One is the kinetic energy density of the bulk motion of particles, *nmv* ^2^/2, in which *n* is the number density, *m* is the mass of particles, and *v* is the bulk velocity. The other is the thermal energy density, which is thermal pressure (*p*_*th*_). On the right‐hand side, the first term represents the change of the U associated with $E_{\|}$, which typically corresponds to the parallel electric field near the X‐line and along separatrices. The second term is the Betatron process due to the conservation of magnetic moment. In this term, the $\frac{\partial B}{\partial t}$ is the magnetic field time derivative, and the ${\vec{v}}_{E}\cdot \nabla B$ is the convective derivative. These two terms adding together result in magnetic field time derivative following the motion of the fluid element, and the calculation can be done in any consistent frame. In our study, both terms are measured in the spacecraft frame. In our calculations, the measurements of the magnetic field and electric field have been averaged and interpolated to the 30 ms time resolution of electrons. The 30 ms is much larger than the gyro‐period of electrons, which was ≲6 ms during the investigated period of this study.

The third term is the Fermi process due to the curvature drift, which occurs during the contraction of the local magnetic field line (Drake, Swisdak, Schoeffler, et al., 2006; Kliem, 1994). In the lowest order of this curvature‐driven Fermi mechanism, the electron's bouncing trajectory follows magnetic field lines. The energy gain of electrons at each bounce is caused by curvature drift along the electric field, corresponding to bouncing off contracting field lines, quantified by the last term in Equation (3).

### 4.2 Curvature Parameter ${Κ}^{2}$ and Theoretical Energy of Adiabatic Electrons

The curvature parameter ${Κ}^{2}$ (Büchner & Zelenyi, 1989) is an important indicator controlling the motion of charged particles in the curved magnetic field lines, especially since this study aims to investigate the dynamics of energetic electrons with energies up to ∼200 keV. The ${Κ}^{2}$ is defined as the ratio of

(4) $${Κ}^{2}=R_{cmin}/r_{\max},$$

where the $R_{cmin}$ is the minimum curvature radius along the curved magnetic field line, which is normally located near the neutral plane. The $r_{\max}$ is the maximum Larmor radius along the magnetic field line of the particles with fixed energies. It is shown that ${Κ}^{2}$ corresponded to the minimum possible ratio of the extreme Larmor to bouncing frequencies of the particle motions (Büchner & Zelenyi, 1989)

(5) $${Κ}^{2}=\Omega_{L,\min}/\omega_{b,\max},$$

where the $\Omega_{L,\min}$ is the possible minimum value of the Larmor frequency. The $\omega_{b,\max}$ is the possible maximum bouncing frequency.

The situation of ${Κ}^{2}$ >> 1 corresponds to the adiabatic case, where the guiding center approximation is valid (see Appendix B on the parallel velocities of particles are much larger than gradient and curvature drifts under guiding center approximation). However, when ${Κ}^{2}$ approaches unity, the particle trajectory would be chaotic due to the resonance of bouncing motion and gyro‐motion. Consequently, strong pitch angle scattering would occur (Büchner & Zelenyi, 1989; Gray & Lee, 1982; Sergeev et al., 1983). Büchner and Zelenyi (1989) shows that as ${Κ}^{2}$ become comparable with 25, the trajectories of particles would start chaotic. Some other studies show that ${Κ}^{2}≲10$ correspond to strong chaotic trajectories (see Propp & Beard, 1984; Sergeev et al., 1983; S. L. Young et al., 2008). The pitch angle scattering causes the distribution of particles to be isotropic.

Here, in the analysis, we introduce the highest energy of adiabatic electrons in theory through

(6) $$W=q^{2}B^{2}R_{cmin}^{2}/2m_{e}{Κ}^{4},$$

where the *q* is the electron charge, the *B* is the magnetic field intensity, and *m*_e_ is the electron mass. Since ${Κ}^{2}≲10$ correspond to strong chaotic trajectories of particles, we introduce $W_{chaotic}$ with ${Κ}^{2}=10$. The electrons with energies higher than $W_{chaotic}$ are in chaotic trajectories and would be subject to strong stochastic dynamics. The particles with energies smaller than $W_{chaotic}$ are quasi‐adiabatic particles.

The local curvature radius of the magnetic field line ${\left(\vec{b}\cdot \nabla \vec{b}\right)}^{-1}$ can be derived through the four spacecraft's simultaneous measurements from the MMS. During the interval from 14:42:45 to 14:44:10 UTC including the flux rope pair and the X‐lines, MMS was located close to the neutral plane with small *B*_*x*_ and as revealed by the G‐S reconstructions shown in Figure 2. Therefore, the calculated curvature radius should be close to the values of the minimum curvature radius of the curved field lines in the plasma sheet.

**Figure 7:**
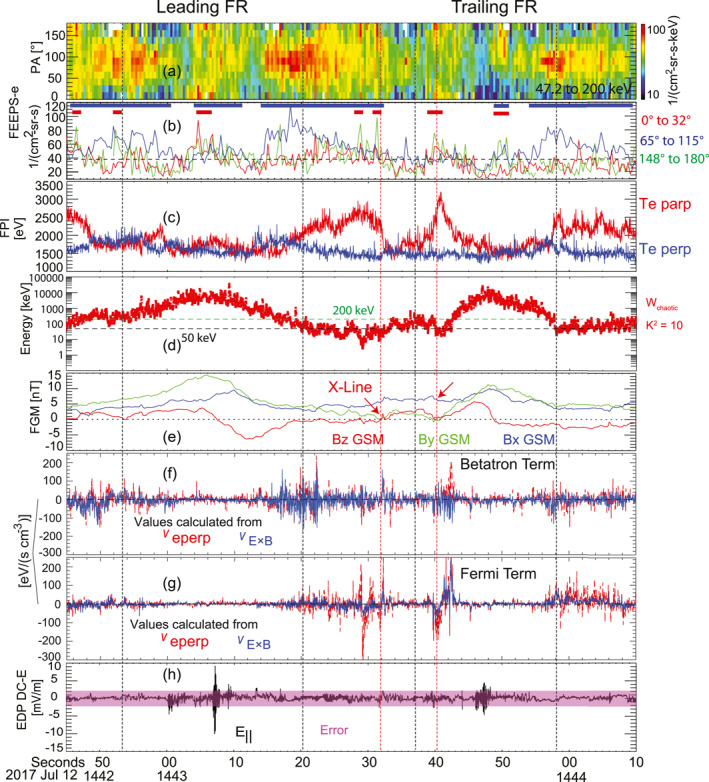
Energization processes of energetic electrons associated with flux ropes and X‐line. Panels (a and b) are from Figures 4d and 4e. (c) Electron temperature from FPI. The parallel temperature is in red and the perpendicular temperature is in blue. (d) The highest energy of adiabatic electrons (*W*_chaotic_), which corresponds to ${Κ}^{2}=10$. The ${Κ}^{2}$ is the ratio of the minimum curvature radius to the maximum Larmor radius of electrons. (e) Magnetic field components, *B*_*x*_ in blue, *B*_*y*_ in green, *B*_*z*_ in red. (f and g) The values of the Betatron and Fermi terms, the intensity of the red line is calculated based on FPI measured electron bulk velocity, the blue line is from the drifting velocity calculated from EDP DC electric field and magnetic field. (h) Parallel electric field (*E*_||_) and errorbar from EDP DC electric field.

**Figure 8:**
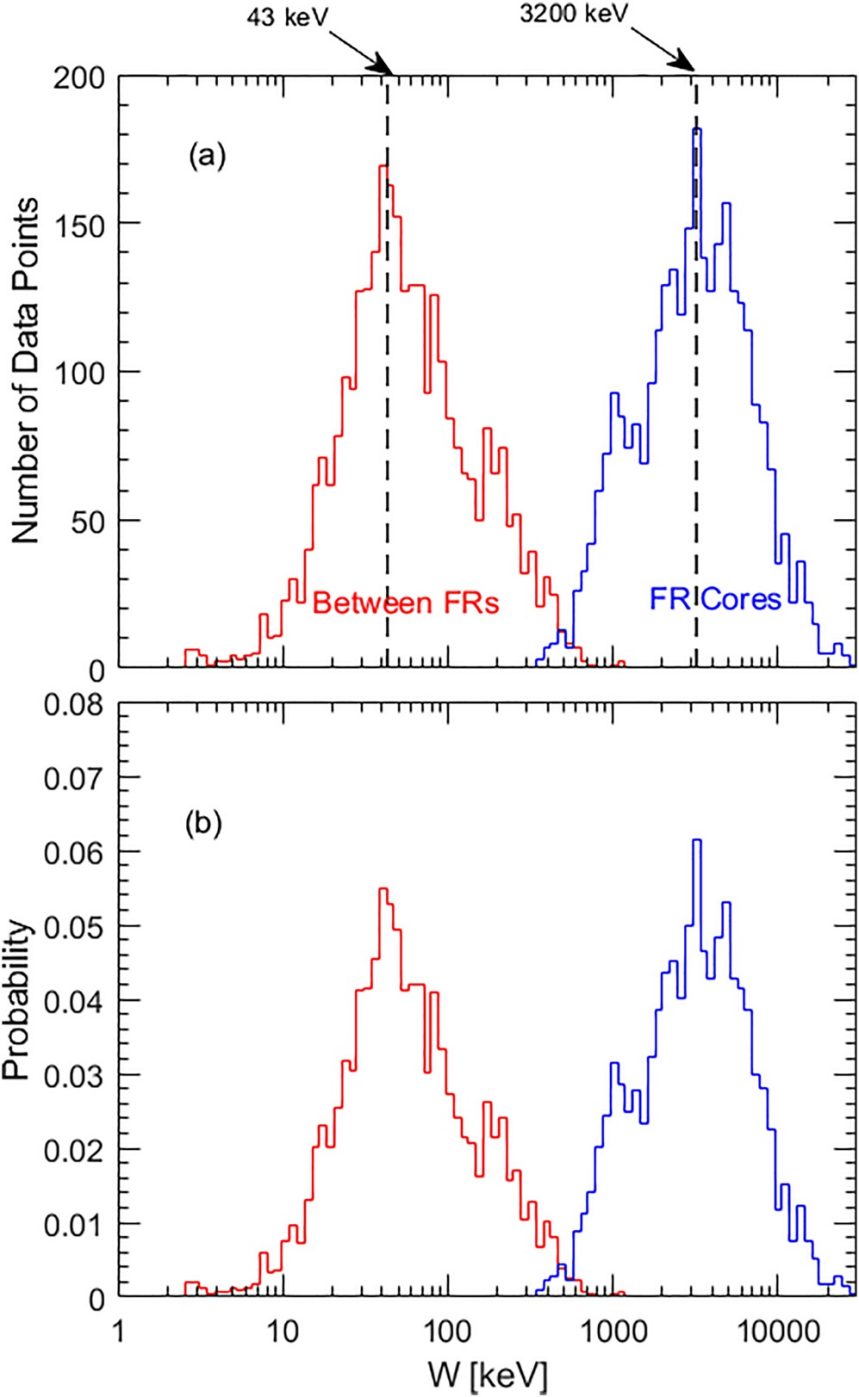
The histograms of the highest energy of adiabatic electrons ($W_{chaotic}$) in the regions between the leading flux rope and trailing flux rope (red lines) and in the flux rope cores (blue lines). The region between the flux rope pair includes the data points from 14:43:17 to 14:43:34. The flux rope cores include the data points from 14:42:59 to 14:43:13 in the leading flux rope, and 14:43:45 to 14:43:53 in the trailing flux rope. (a) The number of data points in each interval. (b) The probability of data points in each interval.

Figure 7d shows the values of $W_{chaotic}$ during the interval between 14:42:45 to 14:44:10 UTC. Figure 8 shows the histograms of the $W_{chaotic}$ in the regions between the rear boundary of the leading flux rope and the front boundary of the trailing flux rope (in red). In the region between the flux rope pair, including the reconnection X‐lines, the $W_{chaotic}$ can be as low as a few keV. The most probable $W_{chaotic}$ was ∼43 keV. Therefore, the energetic electrons with energies from 50 to 200 keV were in chaotic trajectories in the region between the flux rope pair.

Inside the flux ropes, the $W_{\mathrm{chaotic}}$ increased (blue lines in Figure 8). The most probable $W_{chaotic}$ was as high as 3 MeV. This is because the strong helical magnetic field lines in the flux ropes correspond to large curvature radii of the magnetic field lines (e.g., Bergstedt et al., 2020; Shen et al., 2007; Sun et al., 2019; Y. C. Zhang et al., 2013), which in turn results in high $W_{chaotic}$. The electrons in the energy range from ∼50 to 200 keV investigated here were in regular orbit inside the flux ropes.

### 4.3 Betatron and Fermi Processes

Figures 7f and 7g show the values of the Betatron and Fermi processes based on the MMS measurements. The dashed red line and blue line represent the values obtained from the perpendicular component of electron velocity $\left({\vec{v}}_{eperp}\right)$ and drifting velocity due to the electric field $\left({\vec{v}}_{E\times B}\right)$, respectively. The values calculated in the two ways are consistent with each other validating the calculations.

It can be seen that the Betatron and Fermi processes include both acceleration (positive values) and deceleration (negative values) around the flux ropes. The acceleration and deceleration due to the Betatron process corresponded to the compression and expansion of the local magnetic field. The acceleration and deceleration of the Fermi process corresponded to the contraction and stretching of the curved magnetic field lines. The acceleration and deceleration appeared alternately indicating that the magnetic field and plasma processes were quite complex during this interval around the flux rope. The complex plasma and field conditions were likely driven by the frequently appeared X‐lines since several reconnection X‐lines were observed around the two flux ropes. Although the acceleration and the deceleration of particles appear alternately, the enhancements of the energetic electron fluxes indicated that some electrons gained energies from the electromagnetic field.

Another feature is that the Betatron and Fermi processes were stronger on the boundaries of the flux ropes than in the flux rope cores. The values of the Betatron process can be as large as ∼100–200 eV/(s cm^−3^). The strong Betatron process corresponded to the enhanced energetic electrons in the perpendicular direction on the boundaries of the flux ropes. The values of the Fermi process (Figure 7g) were strong near the X‐line regions (the two dashed vertical red lines), which were ∼100–200 eV/(s cm^−3^). Energetic electron beams appeared in the anti‐parallel or/and parallel directions as shown in Figures 7a and 7b. The parallel temperature of electrons measured by FPI with energies lower than 30 keV (Figure 7c) was enhanced around the reconnection X‐lines and the regions where the values of the Fermi process were large.

### 4.4 Parallel Electric Fields Carried by Electrostatic Structures

As shown in Figure 7h, the parallel electric field $\left(E_{\|}\right)$ with intensities larger than the errorbar intermittently appears inside the flux ropes. Two types of structures related with $E_{\|}$ were identified in the flux rope pair. One structure is the double layers (see Block, 1972; Ergun et al., 2001), which correspond to a unipolar profile in $E_{\|}$. The other is the electrostatic solitary waves (ESW) (see Boström et al., 1988; H. S. Fu et al., 2020; Khotyaintsev et al., 2010; Matsumoto et al., 2003), which correspond to a packet of bipolar $E_{\|}$. The ESWs including positive electrostatic potentials are often interpreted as electron holes, and negative electrostatic potentials are ion holes. It is suggested that electron holes are produced in the nonlinear stage of electron‐streaming instabilities (see Omura et al., 1996), while the ion holes are produced by the ion‐streaming instabilities (see Johnsen et al., 1987; R. Wang et al., 2022).

**Figure 9:**
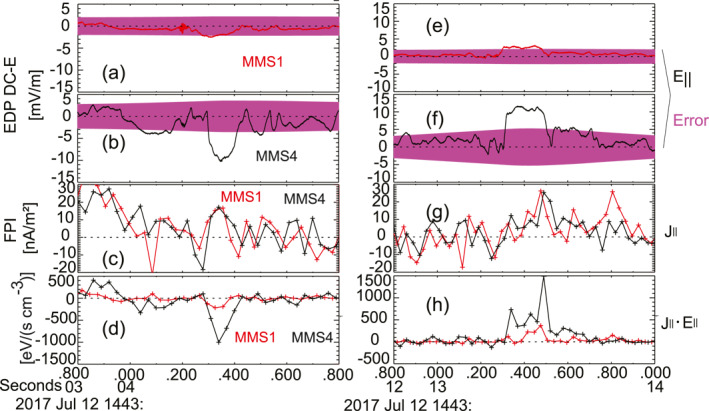
Observations of double layers corresponding to the unipolar parallel electric field in the leading flux rope. (a and e) Parallel electric field $E_{∥}$ from MMS1. (b and f) Parallel electric field $E_{∥}$ from MMS4. (c and g) Field‐aligned currents $J_{∥}$, the current density is calculated from particle moments. (d and h) Energy dissipation rate of $J_{∥}\cdot E_{∥}$. The red and blue lines are the measurements from MMS1 and MMS4, respectively.

Figure 9 shows two examples of the observed double layers. Figure 9b shows two successive double layers with the largest amplitude in unipolar $E_{\|}$ of ∼−10 mV/m. Figure 9d shows that the $E_{\|}J_{\|}$ is negative in both double layers. The peak value for the $E_{\|}J_{\|}$ was ∼−1,100 eV/(s cm^−3^), and the negative value indicated that more particles lost energy to the electromagnetic fields than gained energy from the electromagnetic fields. The traveling speed of the second double layer was calculated to be ∼90 km/s by assuming that it traveled along the magnetic field and the spacecraft distance was projected onto the field. This double layer lasted around ∼0.15 s, and thus, the potential drop $\left(\phi_{\|}=\int E_{\|}vdt\right)$ was calculated to be ∼−100 V. The background *T*_*e*_ was ∼1.8 keV. Therefore, this double layer can contribute to 5.6% of the electron thermal energy.

On the right‐hand side, Figures 9e–9h show another double layer that is observed by MMS1 and MMS4 with an amplitude of ∼12 mV/m. Figure 9h shows that the $E_{\|}J_{\|}$ is positive with a peak value of ∼1,500 eV/(s cm^−3^). The positive values indicated that particles gained energy from the electromagnetic fields. This double layer corresponds to a traveling speed of ∼560 km/s and a potential drop of ∼1 kV, which can contribute to ∼56% of the electron thermal energy.

Based on the deduced propagation speeds and time durations of the double layers, the scales of the double layers along the magnetic field were determined to be ∼14 and ∼67 km, respectively. The electron Debye length was ∼3 km, so the scales of the double layers were larger than the electron Debye length. The separation between MMS1 and MMS4 perpendicular to the magnetic field was ∼12 km. These indicate that the perpendicular scales of the double layers are comparable to 12 km.

**Figure 10:**
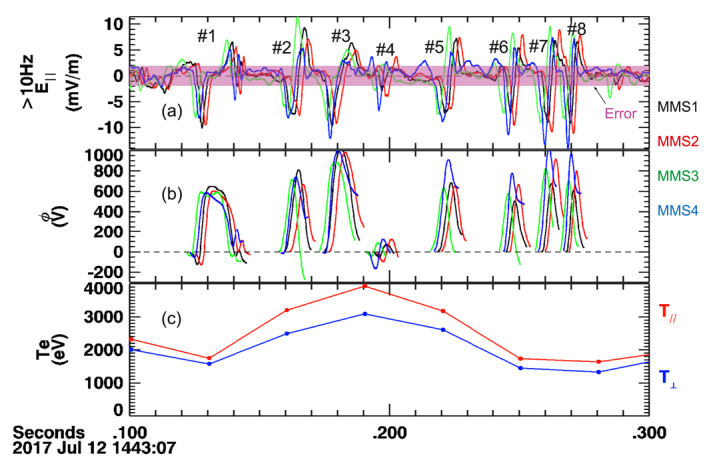
Observations of a packet of electrostatic waves (ESW) between 14:43:07.1 and 14:43:07.3 UTC. (a) Parallel electric field $E_{∥}$ and errorbars. The $E_{∥}$ is filtered at >10 Hz. The $E_{∥}$ errorbar is from MMS1's values. (b) Potential drop associated with the individual $E_{∥}$ bipolar waveform. (c) Electron temperature from FPI.

Figure 10 shows an ESW packet observed inside the leading flux rope between 14:43:07.1 and 14:43:07.3 UT, which includes the measurements of the four spacecraft of the MMS. We identified eight waveforms in this packet. Employing the four‐spacecraft timing analysis, the propagation speeds $\left(v_{ESW}\right)$ of the waveforms were determined to be from 3,400 km/s to 4,900 km/s and they propagated anti‐parallel within 10° from the magnetic field lines. Their propagation properties have been summarized in Table 2. The potential drop associated with each waveform can be calculated through $\Phi_{\|}=\int E_{\|}\left(t\right)v_{ESW}dt$. The potential drops were shown in Figure 10b. Most of the waveforms (7 out of 8) corresponded to positive net potentials, which ranged from 0.1 to 0.65 kV. Therefore, this ESW may be electron holes. The T_e_ was ∼3 keV, and the $\Phi_{\|}$ associated with the ESW waveform can contribute ∼20% of the electron thermal energy.

**Table 2:** Properties of Individual Solitary $E_{\left|\right|}$ Structures in the Electrostatic Wave Packet Shown in Figure 10

Event number	Propagation velocity (km/s)	Propagation angle relative to background magnetic field (degree)	Peak potential (V)	Net potential (V)
#1	4,900 × (−0.52, −0.85, −0.02)	178	650	100
#2	3,600 × (−0.53, −0.85, −0.03)	178	800	340
#3	4,200 × (−0.62, −0.78, −0.09)	172	1,000	550
#4	3,400 × (−0.42, −0.90, −0.10)	173	−170	−50
#5	3,700 × (−0.44, −0.90, −0.05)	174	910	640
#6	4,100 × (−0.43, −0.90, −0.11)	172	770	460
#7	4,100 × (−0.50, −0.87, −0.06)	177	1,020	650
#8	4,700 × (−0.42, −0.91, −0.06)	172	960	590

It is necessary to make a few notes on the ESWs. First, the bipolar structures are predominantly in the parallel component of the electric field, which suggests that the ESWs are quasi 1‐dimensional structures along the magnetic field. Second, their propagating speeds (3,400–4,900 km/s) are several times the background convection speed (HT speed of 600 km/s). The speeds of the satellite were less than 1 km/s during this event. The calculations of the potential drops may be affected by the spatial distribution of the individual bipolar waveform but shall be small since the differences at different satellites are not significant compared to the potential amplitude. Third, the differences between the spacecraft on the $E_{\|}$ may be regarded as the spatial inhomogeneous or temporal variations of the waves. The last note is that electrons may not exhibit clear acceleration features in the ESWs, as shown by H. S. Fu et al. (2020).

### 4.5 Electron Distribution Functions and Parallel Electric Potential

The observed energetic electrons and electron temperature result from an energization history. The full evolution of the electric and magnetic fields of the reconnection X‐lines and flux ropes is impossible to obtain for our case. However, since particles were accelerated/interacted with the electric and magnetic fields, we may be able to derive some information from the measured particles' distributions, similar to the analysis in Egedal et al. (2016) and Wetherton et al. (2021). We examine the electron distribution functions to understand the possible energization processes that worked on the electrons.

**Figure 11:**
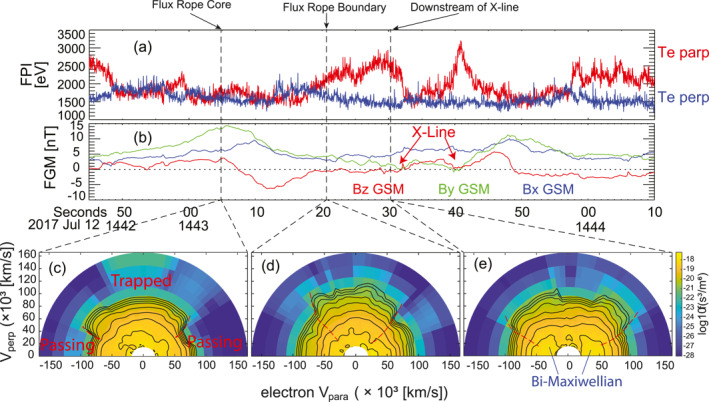
Electron distribution functions measured by FPI (up to 30 keV). (a) Electron temperature measured by FPI, *T*_eperp_ is in blue, *T*_epara_ is in red. (b) Magnetic field components, *B*_*x*_ in blue, *B*_*y*_ in green, *B*_*z*_ in red. (c) Electron distribution functions inside the leading flux rope, (d) on the rear boundary of the leading flux rope, and (e) downstream of an X‐line.

Figure 11 includes three electron distribution functions measured by FPI with energies up to 30 keV. Figure 11c shows an electron distribution function inside the leading flux rope. This electron distribution function includes clear trapped‐passing boundaries near the parallel and anti‐parallel directions. The trapped‐passing boundaries indicate that the trapped electron model due to parallel electric potentials near the reconnection separatrices (Egedal et al., 2012) worked on the electrons. Trapped electrons are subject to pitch angle scatterings. Meanwhile, trapped electrons are heated in the parallel direction. Inside the flux rope, the magnetic field has a significant out‐of‐plane component, that is, the *y* component (Figure 11b). Thus, magnetic field lines are connected to the regions out of the plane of the flux rope. The curvature radii of the magnetic field lines inside the flux rope are significantly larger than the gyro‐radii of the electrons with energies lower than 30 keV, which indicates that the pitch angle scattering does not occur inside the flux rope. Therefore, the features of this electron distribution suggest that the electrons are energized in a place out‐of‐plane of the flux rope. The magnetic field lines in which the electrons gyrate likely connect to reconnection separatrices since the formation of trapped‐passing boundaries in the electron distributions requires parallel electric potentials. This is a clear 3‐D feature of the reconnection and flux ropes.

Figure 11d shows an electron distribution on the rear boundary of the leading flux rope. This electron distribution function includes trapped‐passing boundaries near the parallel and anti‐parallel directions. Meanwhile, electrons are energized in the perpendicular directions. This perpendicular energization in the electron distribution is a typical feature of Betatron acceleration. Similar electron distributions can be seen in Egedal et al. (2012) and S. Wang et al. (2016).

Figure 11e shows an electron distribution function downstream of the reconnection X‐line. It shows clear trapped‐passing boundaries and a bi‐Maxwellian distribution in the parallel and anti‐parallel directions. This fattened electron distribution function indicates strong pitch angle scattering and heating in the parallel and anti‐parallel direction, which is consistent with the trapped electron model (Egedal et al., 2012, 2016). The bi‐Maxwellian suggests that the Fermi‐reflection acceleration occurs as well (Fermi‐reflection acceleration see Drake, Swisdak, Che, and Shay [2006]). The above features clearly indicate that the electron parallel heating attributes to both the trapped electron model and Fermi acceleration.

## 5 Discussions

### 5.1 Energization Mechanisms in Flux Ropes and Between Flux Ropes

This study investigates the energization of electrons associated with a pair of tailward traveling flux ropes and the reconnection X‐lines around it. The flux rope pair corresponds to enhancements of energetic electron fluxes with energies above 45 keV on the boundaries and the core. Four of the five reconnection X‐lines are likely electron‐only reconnection X‐lines. Investigations on the flux rope pair and reconnection X‐lines have revealed energization mechanisms for electrons near the reconnection X‐lines, on the boundaries of the flux ropes, and in the core of flux ropes.

**Figure 12:**
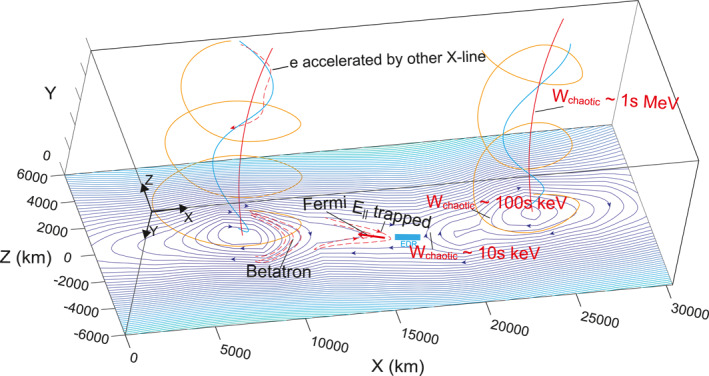
A schematic figure on the structure and energization processes associated with the flux rope pair from this study. Theoretical predicted highest energy of adiabatic electrons (*W*_chaotic_) is a few keV around the X‐lines, tens of keV immediately downstream of the X‐lines, hundreds of keV on the flux rope boundaries, and a few MeV in the flux rope cores. Electrons are accelerated at the reconnection X‐lines. The curvature‐driven Fermi acceleration and the trapped electrons due to parallel electric field occur downstream of the X‐lines. Betatron acceleration is strong on flux rope boundaries. Electrons can be trapped in the regions between the flux rope pair due to mirror force and parallel electric potential. Electrons are accelerated by reconnection X‐lines on the other dawn‐dusk direction in the core of the flux ropes.

Our investigations suggest the following acceleration scenarios, which are summarized in Figure 12. At first, the reconnection X‐lines cause strong energy dissipations $\left(\vec{J}\cdot {\vec{E}}^{\prime }\neq 0\right)$. The positive $\vec{J}\cdot {\vec{E}}^{\prime }$ is observed, which indicates that the electromagnetic energy is converted into the particle's kinetic and thermal energies. However, as we discussed, since MMS was not close enough to the current sheet center, negative $\vec{J}\cdot {\vec{E}}^{\prime }$ is also observed, which is a common feature in the outer EDR (see Daughton et al., 2006; Hwang et al., 2017; Karimabadi et al., 2007; Xiong et al., 2022).

Second, our calculations show that the values of the Fermi acceleration term are strong downstream of the reconnection X‐lines. Electron distribution functions include clear trapped‐passing boundaries and parallel heating, which suggest that the parallel electric field near the reconnection separatrix worked on the electrons. Therefore, the electron parallel heating and the enhancements of the energetic electrons in the parallel/antiparallel directions downstream of the X‐lines are likely a combined result of the Fermi acceleration and the parallel electric potential.

Meanwhile, although the theoretically predicted energy of the adiabatic electrons is limited to tens of keV in this region, energetic electron beams with energy above 50 keV are observed. Since the electrons with energies higher than 50 keV are possibly in a chaotic trajectory, the energetic electron beams shall be generated by other processes. For example, they could be generated by the reconnection electric field, similar to Turner et al. (2021), who reported such high energy electrons near a reconnection X‐line in the magnetotail.

Third, when electrons travel further downstream in the reconnection outflow and pile up on the outer boundaries of the flux ropes. They are subject to strong Betatron acceleration. The values of the Betatron acceleration are strong near the outer boundaries of the flux ropes, where electrons are energized in the perpendicular direction. The theoretically predicted energy of the adiabatic electrons is as high as a few hundred keV.

A fact is that the acceleration and deceleration of the Betatron process appear alternately indicating that the compression and expansion of the local magnetic field appear alternately. The acceleration and deceleration of the Fermi process appear alternately indicating that the electron's convection electric field drift moves along or opposite the magnetic curvature direction alternately. These observations might indicate that the magnetic field and plasma process are turbulent during this interval around the flux rope, which might be driven by the frequently appearing reconnection X‐lines surrounding the flux rope pair. In the turbulent plasma sheet, as shown in Bergstedt et al. (2020) and Ergun et al. (2020a), significant electromagnetic field energy is observed to be converted to particles, however, a large fraction of energy can return from particles to the electromagnetic field.

Fourthly, the values of the Betatron and Fermi processes are both small inside the flux ropes. However, the double layers, which correspond to unipolar $E_{\|}$, and the ESWs, which correspond to a packet of bipolar $E_{\|}$, are observed inside the flux ropes. The potential drop associated with the double layers (∼1 kV) can be as large as half of the electron thermal energy. The ESWs exhibit positive net potentials, and the net potential drops associated with waveforms can contribute to 20% of the electron thermal energy. Therefore, it is hard for these structures to accelerate electrons up to energy above 50 keV. Electron distribution functions inside the flux rope suggest that the magnetic field lines may connect to another X‐line that is out‐of‐plane of the flux rope. Section 5.3 discusses more on what the enhancement of energetic electrons and electron distribution functions inside flux ropes imply. The theoretically predicted energy of the adiabatic electrons can be as high as a few MeV.

In a simulation study, Hoshino et al. (2001) have shown two‐step accelerations of the supra‐thermal electrons (>20 keV) associated with magnetic reconnection, during which electrons are first accelerated by the reconnection electric field and then further are accelerated by the drifts associated with the gradient of the magnetic field and curvature. S. Wang et al. (2016) and Eriksson et al. (2020) have shown the step accelerations of electrons associated with reconnection outflows from the in situ measurements. In the second step of acceleration, Hoshino et al. (2001) have shown that supra‐thermal electrons correspond to a κ value close to unity, and therefore they are subject to strong stochastic accelerations. The observations in our study have confirmed the accelerations of electrons around the reconnection X‐lines are similar to the first‐step acceleration in their scenario. However, our observations of the energetic electrons in the boundaries of the flux ropes, which corresponds to the reconnection outflows, are strongly subject to adiabatic accelerations, that is, the Betatron and Fermi accelerations. The differences could be because our observations are about regions around flux ropes, while the above simulations and observations do not include flux ropes.

### 5.2 Electron Power‐Law Spectra and Fermi‐Reflection Acceleration

This study has investigated the power‐law spectra (κ) of energetic electrons around the reconnection X‐lines and the flux rope pair. The κ values are summarized in Table 3. Electron spectra near the reconnection X‐lines are harder than the electron spectra associated with flux ropes. Electron spectra in the flux rope cores are slightly harder than those spectra on the flux rope boundaries.

**Table 3:** The κ of Electron Power‐Law Spectra Associated With Flux Ropes and Reconnection Diffusion Region From the Measurements of Different Spacecraft in Earth's Plasma Sheet

	Flux ropes	Reconnection diffusion region	Spacecraft
This study	3.54–3.82	∼3.34	MMS
Øieroset et al. (2002)	‐‐‐	2.8–3.7	Wind
Wu et al. (2015)	‐‐‐	∼2.5–4.3	THEMIS
Oka et al. (2016)	‐‐‐	3.0–4.0	THEMIS
Zhou et al. (2016)	‐‐‐	∼2.0–5.0	Cluster
Cohen et al. (2021)	‐‐‐	2.0–5.0	MMS
Huang et al. (2012)	2.24	2.07–2.12	Cluster
L.‐J. Chen et al. (2009)	4–5.3	‐‐‐	Cluster
G. Chen et al. (2019)	‐‐‐	∼3.3	Cluster
H. S. Fu et al. (2019)	‐‐‐	4.0–5.0	Cluster
Zhong et al. (2020)	3.3–3.6	‐‐‐	MMS

The accelerations due to Fermi‐reflection have been investigated by simulations. In the scenario of Fermi reflection suggested by Drake, Swisdak, Che, and Shay (2006), electrons bounce between the high latitudes of flux ropes or are trapped in the closed field lines of flux ropes. However, Drake, Swisdak, Che, and Shay (2006) described a two‐dimensional picture.

In our study, the flux ropes are 3‐dimensional structures. Flux ropes include helical field lines with a component in the out‐of‐plane, that is, *y*, direction, which are not closed field line loops. Therefore, electrons are unlikely trapped in the closed field lines in 3‐dimensional. However, electrons can still be trapped in the regions between the flux rope pair. Electrons can be reflected by the mirror force during their travel to the high latitude of the flux ropes since the magnetic field intensity is much higher on the high latitude than in the reconnecting current sheet. Electrons may also be trapped by the parallel potential drop.

The in situ measurements in our study have revealed a few features of the acceleration of electrons, which are not the same as the 2‐dimensional structures in the simulation. First, electron spectra around the reconnection X‐lines between the flux rope pair correspond to the hardest spectra. The theoretical predicted highest energy of adiabatic electrons near the reconnection X‐lines is only a few keV. This indicates that electrons of higher energy (>40 keV) are in a chaotic trajectory near the reconnection X‐lines. The hardest spectra can be due to non‐adiabatic accelerations associated with the reconnection X‐lines.

Second, the curvature‐driven Fermi process appears to be strong downstream of the X‐line but is small inside the flux rope from our in situ measurements, while the Fermi process seems strong both downstream of the X‐line and inside the flux rope in simulations.

Third, strong Betatron acceleration, that is, the pileup of magnetic field lines, is found downstream of the X‐lines and on the flux rope boundaries. The enhancement of energetic electrons in the perpendicular direction does imply the significant role of Betatron acceleration in accelerating electrons. Since the magnetic field intensity is high and the curvature radius of the magnetic field line is large on the high latitude of the flux ropes. Electrons traveling from the equatorial plane to the high latitude could be forced back by the mirror force and then trapped in the field lines. The parallel electric potential suggested by Egedal et al. (2015) may also support trapping the electrons. One note is that the energetic electron fluxes increased from the X‐line to the flux rope boundaries. The softer spectrum on the boundaries of the flux ropes is associated with the enhancement of fluxes at tens of keV, indicating the energization at such energy ranges.

Many studies have investigated the power‐law spectra of energetic electrons associated with reconnection diffusion regions and flux ropes. Here we make a comparison with those results. It needs to note that these studies are using different quantities to derive the index of power‐law spectra, including differential particle flux, and phase space density. This study used differential energy flux. To make the comparison, we have transformed all of those indices to make them consistent with the κ used in this study (see Oka et al. [2018] for detail on how to make the transform). The results are summarized in Table 3.

The values of the κ associated with the flux ropes obtained in this study are similar to those in Zhong et al. (2020), which used MMS measurements. However, the values of the κ obtained by Cluster measurements were broader, that is, from 4 to 5.3 in L.‐J. Chen et al. (2009), and 2.24 in Huang et al. (2012). The values of the κ around the reconnection X‐lines obtained in this study are similar or located between those values obtained by Wind (Øieroset et al., 2002), Cluster (G. Chen et al., 2019; H. S. Fu et al., 2019; Huang et al., 2012; Zhou et al., 2016), THEMIS (Oka et al., 2016; Wu et al., 2015) or MMS (Cohen et al., 2021).

Many simulations have produced the power‐law spectra of electrons associated with the reconnection X‐lines and flux ropes. Here, we compare with Q. Zhang et al. (2021), which have demonstrated that the curvature‐driven Fermi mechanism can efficiently accelerate electrons and develop power‐law spectra with an index of ∼2.8. Q. Zhang et al. (2021) have employed 3‐dimensional fully kinetic simulations. In their simulations, the guide field is weak (0.2), which is generally true of Earth's magnetotail. Several flux ropes are generated in their simulations. The distribution of electrons is averaged over the entire domain region of their simulations. The value of 2.8 is smaller than our observations (3.3–3.8), which can attribute to the strong turbulent plasma in their simulations.

### 5.3 What Do Enhancements of Energetic Electrons Inside Flux Ropes Imply?

The electrons demonstrate several types of distributions associated with the two flux ropes and reconnection X‐lines. The energetic electron fluxes are enhanced on the boundaries and in the core of the flux ropes. The perpendicular enhanced energetic electrons are observed on the boundaries of the flux ropes, which has been suggested due to the local acceleration of the Betatron process. However, the values of the local Betatron and Fermi terms are small in the core of flux ropes, which could not account for the enhancements of energetic electrons in those regions. The double layers and ESWs observed inside flux ropes can contribute to around one keV energy for the electrons, which is difficult to accelerate the electrons to energies above 50 keV.

Magnetic flux ropes consist of helical magnetic field lines. In the flux rope core, the magnetic field lines are significant in the dawn or dusk, that is, *y* in GSM, direction since the *B*_*y*_ is the dominant component for our cases. The energetic electrons shall be accelerated in another dawn or dusk direction other than the *X*‐*Z* plane crossed by the spacecraft and then transport into the flux rope core, that is, a 3‐dimensional effect. This scenario is supported by the electron distribution functions near the flux rope core, which contain clear trapped‐passing boundaries in the parallel/anti‐parallel directions and clear evidence that the electrons are accelerated by parallel electric potentials near the reconnection separatrices and the magnetic field lines are connecting to an X‐line in another dawn or dusk direction.

Another implication is related to the acceleration mechanism for ∼200 keV to several MeV electrons observed in the deep tail (downtail ∼100 *R*_*E*_) (Krimigis & Sarris, 1980; Richardson et al., 1996; Slavin et al., 1992). These studies all observed energetic electrons in the Earth's magnetotail during substorms ranging in energy from ∼200 keV injection events in the near‐tail to several MeV at distances of ∼30 *R*_*E*_ to 100 *R*_*E*_ tailward of the Earth. Slavin et al. (1992) studied an interval of ∼36 hr in duration when ISEE‐3 was between 76 and 80 *R*_*E*_ tailward of the Earth. They observed 12 isolated, quasi‐periodic substorms that first loaded and then unloaded energy stored in the lobes through the formation and ejection of a series of plasmoids. Each substorm produced energetic electrons with energies up to ∼200 keV in the near‐tail. Later, Richardson et al. (1996) analyzed ISEE‐3 and IMP‐8 energetic electron measurements and found that most of the ISEE‐3 substorm events were associated with energetic electron events, ∼200 keV to 2 MeV, in the middle to deep magnetotail (i.e., ∼30 to 100 *R*_*E*_). Richardson et al. (1996) observed a few of these highly energetic electron events beyond 100 R_E_ downstream despite ISEE‐3's apogee of ∼240 *R*_*E*_. Our study implies that the electrons can remain adiabatic up to MeV in the flux rope core. In the regions near reconnection X‐lines and flux rope boundaries, the highest energy of adiabatic electrons is a few hundred keV or lower, electrons would be escaped before reaching the energy of MeV. Inside the flux ropes, the helical magnetic field lines correspond to a large curvature radius and therefore higher adiabatic energy of electrons. However, the local acceleration terms do not manifest large values inside the flux ropes observed in this study. Since the flux rope pair is located in the near‐Earth tail (downtail ∼24.5 *R*_*E*_), and observations of MeV electrons in Richardson et al. (1996) and Slavin et al. (1992) are in the deep tail, electrons may be accelerated further as the flux rope traveling further downtail. Especially, merging flux ropes might prevent the escape of energetic electrons and enhance the accelerations of electrons as suggested by Pritchett (2008), Oka et al. (2010), and many others.

## 6 Conclusions

This study has investigated the properties and accelerations of electrons up to 200 keV associated with a flux rope pair and a few reconnection X‐lines. The Grad‐Shafranov (GS) reconstruction reveals that the MMS spacecraft cross near the center of the flux ropes, and both flux ropes have elongated profiles with scales of ∼10 ion inertial lengths. Five reconnection X‐lines are identified around the flux rope pair. The reconnection X‐lines are accompanied by reversals in electron bulk velocity, strong electric fields, strong current densities, and strong energy dissipations $\left(\vec{J}\cdot {\vec{E}}^{\prime }\right)$. Four of the five reconnection X‐lines are likely electron‐only reconnection X‐lines.

The flux ropes correspond to enhancements of energetic electron fluxes on the boundaries and in the core. Energetic electrons are intense in the directions perpendicular to the local magnetic field on the outer boundaries and in the core of flux ropes. Energetic electron beams are observed as well. The beams appear around the reconnection X‐lines and in the flux rope core. The parallel electron temperature exhibits several peaks with intensities higher than the perpendicular temperature downstream of the reconnection X‐line. The differential energy flux of energetic electrons is fitted by the power‐law distribution. The spectra of energetic electrons around the reconnection X‐lines are the hardest, and the spectra near the flux rope cores are slightly harder than those spectra on the flux rope boundaries.

We have calculated the theoretical predicted highest energy of adiabatic electrons $\left(W_{chaotic}\right)$. Electrons with energies higher than $W_{chaotic}$ are in chaotic trajectories. The energy of the adiabatic electrons is only a few keV around the reconnection X‐lines and is often below 50 keV in the immediately downstream region of the X‐lines. The energy of adiabatic electrons increases to a few hundred keV on the boundaries of flux ropes, and in the core of the flux ropes, the energy of adiabatic electrons can be a few MeV.

Our investigations reveal different energization mechanisms for electrons near the reconnection X‐lines and the flux ropes. The reconnection X‐lines cause strong energy dissipation $\left(\vec{J}\cdot {\vec{E}}^{\prime }>0\right)$, which indicates that the electromagnetic energy is converted into electron kinetic and thermal energies. In the region between the flux rope pair, downstream of the reconnection X‐line, the curvature‐driven Fermi acceleration is strong. The electron distribution functions including clear trapped‐passing boundaries and heating in the parallel direction indicate that electrons are trapped and accelerated by the parallel electric field potential as well. Both curvature‐driven Fermi acceleration and trapped electrons model can account for the enhanced electron parallel temperature and energetic electrons with energy below 50 keV. Since the energetic electrons (>50 keV) are in a chaotic trajectory in this region and the energetic electron beams should not be due to the Fermi process but to other processes, possibly the reconnecting electric field. The Betatron process is strong near the outer boundaries of the flux ropes, which accounts for the enhanced energetic electrons in the perpendicular direction. We propose that electrons can be bounced between the south and north high latitude boundaries of flux ropes by the mirror force and parallel electric field potentials. They would be repeatedly accelerated by the processes occurring in the regions between the flux rope pair.

Furthermore, the acceleration and deceleration of Betatron and Fermi processes appear alternately indicating that the magnetic field and plasma process are turbulent during this interval around the flux rope, which might be driven by the frequently appearing reconnection X‐lines surrounding the flux ropes.

Near the flux rope cores, both Betatron and Fermi processes are weak. Electrostatic structures carry parallel electric fields, which correspond to potential drops smaller than the electron thermal energy and far smaller than the energy of energetic electrons (>50 keV). Because the flux rope cores contain strong core fields, which form helical field lines, we propose that the energetic electrons in the core are accelerated in other dawn‐dusk directions and are transported into the flux ropes. This 3‐dimensional effect is confirmed by the appearance of clear trapped‐passing boundaries in the electron distribution functions in the flux rope core.

## Supporting information

Supporting Information S1

## Figures and Tables

**Figure A1:**
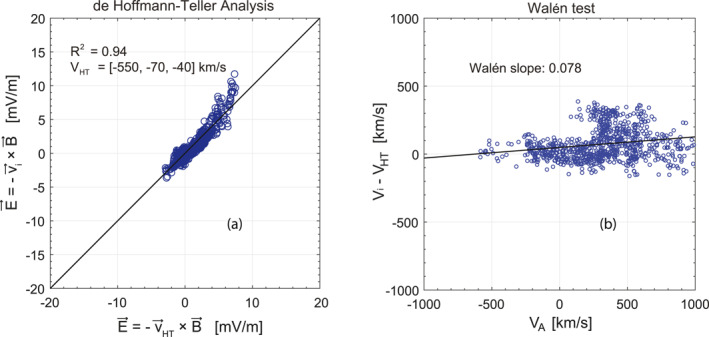
The de Hoffmann‐Teller analysis and the Walén test for the flux rope pair from 14:42:45 to 14:44:10 UTC. (a) Scatter plot of the electric field under the de Hoffmann‐Teller reference frame versus the convection electric field of the ion bulk velocity. The de Hoffmann‐Teller velocity is determined to be (−550, −70, −40) km/s with a correlation coefficient *R* ^2^ of around 0.94. (b) Scatter plot for the ion velocity under de Hoffmann‐Teller frame versus the local measured Alfvén speeds. The regression line, that is, Walén slope, is determined to be around 0.078.

## Data Availability

The data presented in this paper are the L2 data of MMS and can be accessed from MMS Science Data Center (https://lasp.colorado.edu/mms/sdc/public/).

## Appendix A de Hoffmann‐Teller Analysis and Walén Test

The frame of reference of the flux rope pair was determined by the de Hoffmann‐Teller analysis (HT) (Khrabrov & Sonnerup, 1998). Figure A1a includes the scatter plot of the electric field under the de Hoffmann‐Teller reference frame versus the convection electric field of the ion bulk velocity. The correlation coefficient *R* ^2^ is determined to be ∼0.94. The HT frame was determined to be (−550, −70, −40) km/s, corroborating the strong anti‐planetward flow embedding the flux rope pair.

Figure A1b includes the Walén test (Paschmann, 1985; Sonnerup & Guo, 1996). The slope of the regression line in the Walén test is determined to be 0.078. The small slope means that the remaining ion flow speed in the HT frame is much smaller than the Alfvén speed, which indicates the high coherence of the structure and a small magnitude of the remaining flow speed.

## Appendix B Guiding Center Approximation: Parallel Velocities of Particles Are Much Larger Than Gradient and Curvature Drifts During Fermi Acceleration

The guiding center approximation implies that the gyro‐radius of particles is much smaller than the curvature radius.

(B1)
$$R_{c}/r_{Larmor}\gg 1,$$

where the $R_{c}$ is the curvature radius of the curved magnetic field line, and the $r_{Larmor}$ is the Larmor radius of particles. Since $r_{Larmor}=mv_{⊥}/qB$, where m, $v_{⊥}$, and q are the mass, perpendicular velocity, and charge of a particle, we then have

(B2)
$${qBR}_{c}/mv_{⊥}\gg 1.$$

The guiding center approximation also implies that the Larmor frequency is much larger than the bouncing frequency.

(B3)
$$\Omega_{L}/\omega_{b}\gg 1,$$

where the $\Omega_{L}$ is the Larmor frequency, qB/m. The bounce frequency at the curved field line, $\omega_{b}∼2\pi /\left(2\pi R_{c}/v_{∥}\right)=v_{∥}/R_{c}$, where $v_{∥}$ is the parallel velocity of a particle. Thus, Equation B3 can be written as

(B4)
$${qBR}_{c}/mv_{\|}\gg 1.$$

When the magnetic field is inhomogeneous, the gradient of the magnetic field causes gradient drift and the curved field lines cause curvature drift,

(B5)
$$\left\{\begin{array}{c} {\vec{v}}_{\nabla }=\frac{mv_{⊥}^{2}}{2qB^{3}}\vec{B}\times \nabla B\\ {\vec{v}}_{c}=\frac{mv_{∥}^{2}}{qR_{c}^{2}B^{2}}{\vec{R}}_{c}\times \vec{B} \end{array}\right.$$

(Baumjohann & Treumann, 2012). For simplicity, here we do a scaling/dimensional analysis. The curvature and gradient drifts can be written in similar formats,

(B6)
$$\left\{\begin{array}{c} v_{\nabla }\;∼\;\frac{mv_{⊥}^{2}}{2qR_{c}B}\\ v_{c}\;∼\;\frac{mv_{\|}^{2}}{qR_{c}B} \end{array}\right..$$

If we compare the values of the drift velocity with the velocity of particles along the field lines ($v_{∥}$), one can find that

(B7)
$$v_{∥}/v_{\nabla }\;∼\;\frac{2qR_{c}Bv_{∥}}{mv_{⊥}^{2}}.$$

In combination with Equation B2, it shows that those particles with $\left|{2v}_{∥}/v_{⊥}\right|≳1$ correspond to $v_{∥}/v_{\nabla }\gg 1$. Most of the particles satisfy this requirement except a minority with pitch angles close to 90°. This is especially true since Fermi acceleration mostly boosts $v_{∥}$ (Drake, Swisdak, Che, & Shay, 2006).

Similarly,

(B8)
$$v_{∥}/v_{c}\;∼\;\frac{qR_{c}B}{mv_{\|}}.$$

From Equation B4, therefore, $v_{∥}/v_{c}\gg 1$.

Therefore, under guiding center approximation parallel velocities of particles are much larger than gradient and curvature drifts for most particles during Fermi acceleration, so these particles are approximately traveling along the field lines.

## References

[jgra57548-bib-0001] Akhavan‐Tafti, M. , Slavin, J. A. , Sun, W. J. , Le, G. , & Gershman, D. J. (2019). MMS observations of plasma heating associated with FTE growth. Geophysical Research Letters, 46(22), 12654–12664. 10.1029/2019gl084843

[jgra57548-bib-0002] Arnold, H. , Drake, J. F. , Swisdak, M. , Guo, F. , Dahlin, J. T. , Chen, B. , et al. (2021). Electron acceleration during macroscale magnetic reconnection. Physical Review Letters, 126(13), 135101. 10.1103/physrevlett.126.135101 33861105

[jgra57548-bib-0003] Baumjohann, W. , & Treumann, R. A. (2012). Basic space plasma physics (Revised ed.). Imperial College Press.

[jgra57548-bib-0004] Bergstedt, K. , Ji, H. , Jara‐Almonte, J. , Yoo, J. , Ergun, R. E. , & Chen, L.‐J. (2020). Statistical properties of magnetic structures and energy dissipation during turbulent reconnection in the Earth's magnetotail. Geophysical Research Letters, 47(19), e2020GL088540. 10.1029/2020gl088540

[jgra57548-bib-0005] Blake, J. B. , Mauk, B. H. , Baker, D. N. , Carranza, P. , Clemmons, J. H. , Craft, J. , et al. (2016). The fly’s eye energetic particle spectrometer (FEEPS) sensors for the magnetospheric multiscale (MMS) mission. Space Science Reviews, 199(1), 309–329. 10.1007/s11214-015-0163-x

[jgra57548-bib-0006] Block, L. P. (1972). Potential double layers in the ionosphere. Cosmic Electrodynamics, 3, 349.

[jgra57548-bib-0007] Boström, R. , Gustafsson, G. , Holback, B. , Holmgren, G. , Koskinen, H. , & Kintner, P. (1988). Characteristics of solitary waves and weak double layers in the magnetospheric plasma. Physical Review Letters, 61(1), 82–85. 10.1103/physrevlett.61.82 10038699

[jgra57548-bib-0008] Büchner, J. , & Zelenyi, L. M. (1989). Regular and chaotic charged particle motion in magnetotaillike field reversals: 1. Basic theory of trapped motion. Journal of Geophysical Research, 94(A9), 11821–11842. 10.1029/ja094ia09p11821

[jgra57548-bib-0009] Burch, J. L. , Moore, T. E. , Torbert, R. B. , & Giles, B. L. (2016). Magnetospheric multiscale overview and science objectives. Space Science Reviews, 199(1), 5–21. 10.1007/s11214-015-0164-9

[jgra57548-bib-0010] Burch, J. L. , Torbert, R. B. , Phan, T. D. , Chen, L.‐J. , Moore, T. E. , Ergun, R. E. , et al. (2016). Electron‐scale measurements of magnetic reconnection in space. Science, 352(6290), aaf2939. 10.1126/science.aaf2939 27174677

[jgra57548-bib-0011] Chen, G. , Fu, H. S. , Zhang, Y. , Li, X. , Ge, Y. S. , Du, A. M. , et al. (2019). Energetic electron acceleration in unconfined reconnection jets. The Astrophysical Journal, 881(1), L8. 10.3847/2041-8213/ab3041

[jgra57548-bib-0012] Chen, L.‐J. , Bessho, N. , Lefebvre, B. , Vaith, H. , Asnes, A. , Santolik, O. , et al. (2009). Multispacecraft observations of the electron current sheet, neighboring magnetic islands, and electron acceleration during magnetotail reconnection. Physics of Plasmas, 16(5), 056501. 10.1063/1.3112744

[jgra57548-bib-0013] Chen, L. J. , Bhattacharjee, A. , Puhl‐Quinn, P. A. , Yang, H. , Bessho, N. , Imada, S. , et al. (2007). Observation of energetic electrons within magnetic islands. Nature Physics, 4(1), 19–23. 10.1038/nphys777

[jgra57548-bib-0014] Christon, S. P. , Williams, D. J. , Mitchell, D. G. , Huang, C. Y. , & Frank, L. A. (1991). Spectral characteristics of plasma sheet ion and electron populations during disturbed geomagnetic conditions. Journal of Geophysical Research, 96(A1), 1–22. 10.1029/90ja01633

[jgra57548-bib-0015] Cohen, I. J. , Turner, D. L. , Mauk, B. H. , Bingham, S. T. , Blake, J. B. , Fennell, J. F. , & Burch, J. L. (2021). Characteristics of energetic electrons near active magnetotail reconnection sites: Statistical evidence for local energization. Geophysical Research Letters, 48(1), e2020GL090087. 10.1029/2020gl090087

[jgra57548-bib-0016] Dahlin, J. T. , Drake, J. F. , & Swisdak, M. (2014). The mechanisms of electron heating and acceleration during magnetic reconnection. Physics of Plasmas, 21(9), 092304. 10.1063/1.4894484

[jgra57548-bib-0017] Daughton, W. , Scudder, J. , & Karimabadi, H. (2006). Fully kinetic simulations of undriven magnetic reconnection with open boundary conditions. Physics of Plasmas, 13(7), 072101. 10.1063/1.2218817

[jgra57548-bib-0018] Drake, J. F. , Cassak, P. A. , Shay, M. A. , Swisdak, M. , & Quataert, E. (2009). A magnetic reconnection mechanism for ion acceleration and abundance enhancements in impulsive flares. The Astrophysical Journal, 700(1), L16–L20. 10.1088/0004-637x/700/1/l16

[jgra57548-bib-0019] Drake, J. F. , Swisdak, M. , Cattell, C. , Shay, M. A. , Rogers, B. N. , & Zeiler, A. (2003). Formation of electron holes and particle energization during magnetic reconnection. Science, 299(5608), 873–877. 10.1126/science.1080333 12574625

[jgra57548-bib-0020] Drake, J. F. , Swisdak, M. , Che, H. , & Shay, M. A. (2006). Electron acceleration from contracting magnetic islands during reconnection. Nature, 443(7111), 553–556. 10.1038/nature05116 17024088

[jgra57548-bib-0021] Drake, J. F. , Swisdak, M. , Schoeffler, K. M. , Rogers, B. N. , & Kobayashi, S. (2006). Formation of secondary islands during magnetic reconnection. Geophysical Research Letters, 33(13), L13105. 10.1029/2006gl025957

[jgra57548-bib-0022] Egedal, J. , Daughton, W. , & Le, A. (2012). Large‐scale electron acceleration by parallel electric fields during magnetic reconnection. Nature Physics, 8(4), 321–324. 10.1038/nphys2249

[jgra57548-bib-0023] Egedal, J. , Daughton, W. , Le, A. , & Borg, A. L. (2015). Double layer electric fields aiding the production of energetic flat‐top distributions and superthermal electrons within magnetic reconnection exhausts. Physics of Plasmas, 22(10), 101208. 10.1063/1.4933055

[jgra57548-bib-0024] Egedal, J. , Wetherton, B. , Daughton, W. , & Le, A. (2016). Processes setting the structure of the electron distribution function within the exhausts of anti‐parallel reconnection. Physics of Plasmas, 23(12), 122904. 10.1063/1.4972135

[jgra57548-bib-0025] Ergun, R. E. , Ahmadi, N. , Kromyda, L. , Schwartz, S. J. , Chasapis, A. , Hoilijoki, S. , et al. (2020a). Observations of particle acceleration in magnetic reconnection–driven turbulence. The Astrophysical Journal, 898(2), 154. 10.3847/1538-4357/ab9ab6

[jgra57548-bib-0026] Ergun, R. E. , Ahmadi, N. , Kromyda, L. , Schwartz, S. J. , Chasapis, A. , Hoilijoki, S. , et al. (2020b). Particle acceleration in strong turbulence in the Earth’s magnetotail. The Astrophysical Journal, 898(2), 153. 10.3847/1538-4357/ab9ab5

[jgra57548-bib-0027] Ergun, R. E. , Su, Y. J. , Andersson, L. , Carlson, C. W. , McFadden, J. P. , Mozer, F. S. , et al. (2001). Direct observation of localized parallel electric fields in a space plasma. Physical Review Letters, 87(4), 045003. 10.1103/physrevlett.87.045003 11461625

[jgra57548-bib-0028] Ergun, R. E. , Tucker, S. , Westfall, J. , Goodrich, K. A. , Malaspina, D. M. , Summers, D. , et al. (2016). The axial double probe and fields signal processing for the MMS mission. Space Science Reviews, 199(1–4), 167–188. 10.1007/s11214-014-0115-x

[jgra57548-bib-0029] Eriksson, E. , Vaivads, A. , Alm, L. , Graham, D. B. , Khotyaintsev, Y. V. , & André, M. (2020). Electron acceleration in a magnetotail reconnection outflow region using magnetospheric multiscale data. Geophysical Research Letters, 47(1), e2019GL085080. 10.1029/2019gl085080

[jgra57548-bib-0030] Espinoza, C. M. , Stepanova, M. , Moya, P. S. , Antonova, E. E. , & Valdivia, J. A. (2018). Ion and electron κ distribution functions along the plasma sheet. Geophysical Research Letters, 45(13), 6362–6370. 10.1029/2018gl078631

[jgra57548-bib-0031] Fu, H. S. , Chen, F. , Chen, Z. Z. , Xu, Y. , Wang, Z. , Liu, Y. Y. , et al. (2020). First measurements of electrons and waves inside an electrostatic solitary wave. Physical Review Letters, 124(9), 095101. 10.1103/PhysRevLett.124.095101 32202894

[jgra57548-bib-0032] Fu, H. S. , Khotyaintsev, Y. V. , Vaivads, A. , Retinò, A. , & André, M. (2013). Energetic electron acceleration by unsteady magnetic reconnection. Nature Physics, 9(7), 426–430. 10.1038/nphys2664

[jgra57548-bib-0033] Fu, H. S. , Xu, Y. , Vaivads, A. , & Khotyaintsev, Y. V. (2019). Super‐efficient electron acceleration by an isolated magnetic reconnection. The Astrophysical Journal, 870(2), L22. 10.3847/2041-8213/aafa75

[jgra57548-bib-0034] Fu, X. R. , Lu, Q. M. , & Wang, S. (2006). The process of electron acceleration during collisionless magnetic reconnection. Physics of Plasmas, 13(1), 012309. 10.1063/1.2164808

[jgra57548-bib-0035] Gershman, D. J. , Avanov, L. A. , Boardsen, S. A. , Dorelli, J. C. , Gliese, U. , Barrie, A. C. , et al. (2017). Spacecraft and instrument photoelectrons measured by the dual electron spectrometers on MMS. Journal of Geophysical Research: Space Physics, 122(11), 11548–11558. 10.1002/2017JA024518

[jgra57548-bib-0036] Gray, P. C. , & Lee, L. C. (1982). Particle pitch angle diffusion due to nonadiabatic effects in the plasma sheet. Journal of Geophysical Research, 87(A9), 7445–7452. 10.1029/ja087ia09p07445

[jgra57548-bib-0037] Hasegawa, H. , Sonnerup, B. U. Ö. , Klecker, B. , Paschmann, G. , Dunlop, M. W. , & Rème, H. (2005). Optimal reconstruction of magnetopause structures from Cluster data. Annals of Geophysics, 23(3), 973–982. 10.5194/angeo-23-973-2005

[jgra57548-bib-0038] Hau, L. N. , & Sonnerup, B. U. Ö. (1999). Two‐dimensional coherent structures in the magnetopause: Recovery of static equilibria from single‐spacecraft data. Journal of Geophysical Research, 104(A4), 6899–6917. 10.1029/1999ja900002

[jgra57548-bib-0039] Hones, E. W., Jr. (1984). Plasma sheet behavior during substorms. In E. W. Hones Jr. (Ed.), Magnetic Reconnection in Space and Laboratory Plasmas (Ed.) (Vol. 30, pp. 178–184). American Geophysical Union.

[jgra57548-bib-0040] Hoshino, M. (2012). Stochastic particle acceleration in multiple magnetic islands during reconnection. Physical Review Letters, 108(13), 135003. 10.1103/PhysRevLett.108.135003 22540708

[jgra57548-bib-0041] Hoshino, M. , Mukai, T. , Terasawa, T. , & Shinohara, I. (2001). Suprathermal electron acceleration in magnetic reconnection. Journal of Geophysical Research, 106(A11), 25979–25997. 10.1029/2001ja900052

[jgra57548-bib-0042] Hu, Q. , & Sonnerup, B. U. Ö. (2002). Reconstruction of magnetic clouds in the solar wind: Orientations and configurations. Journal of Geophysical Research, 107(A7), SSH 10‐11–SSH 10‐15. 10.1029/2001ja000293

[jgra57548-bib-0043] Hu, Q. , & Sonnerup, B. U. Ö. (2003). Reconstruction of two‐dimensional structures in the magnetopause: Method improvements. Journal of Geophysical Research, 108(A1), SMP 9‐1–SMP 9‐9. 10.1029/2002ja009323

[jgra57548-bib-0044] Huang, S. Y. , Vaivads, A. , Khotyaintsev, Y. V. , Zhou, M. , Fu, H. S. , Retinò, A. , et al. (2012). Electron acceleration in the reconnection diffusion region: Cluster observations. Geophysical Research Letters, 39(11), L11103. 10.1029/2012gl051946

[jgra57548-bib-0045] Hwang, K.‐J. , Sibeck, D. G. , Choi, E. , Chen, L.‐J. , Ergun, R. E. , Khotyaintsev, Y. , et al. (2017). Magnetospheric multiscale mission observations of the outer electron diffusion region. Geophysical Research Letters, 44(5), 2049–2059. 10.1002/2017gl072830

[jgra57548-bib-0046] Ieda, A. , Machida, S. , Mukai, T. , Saito, Y. , Yamamoto, T. , Nishida, A. , et al. (1998). Statistical analysis of the plasmoid evolution with Geotail observations. Journal of Geophysical Research, 103(A3), 4453–4465. 10.1029/97ja03240

[jgra57548-bib-0047] Imada, S. , Nakamura, R. , Daly, P. W. , Hoshino, M. , Baumjohann, W. , Mühlbachler, S. , et al. (2007). Energetic electron acceleration in the downstream reconnection outflow region. Journal of Geophysical Research, 112(A3), A03202. 10.1029/2006ja011847

[jgra57548-bib-0048] Jiang, K. , Huang, S. Y. , Yuan, Z. G. , Deng, X. H. , Wei, Y. Y. , Xiong, Q. Y. , et al. (2021). Statistical properties of current, energy conversion, and electron acceleration in flux ropes in the terrestrial magnetotail. Geophysical Research Letters, 48(11), e2021GL093458. 10.1029/2021gl093458

[jgra57548-bib-0049] Johnsen, H. , Pécseli, H. L. , & Trulsen, J. (1987). Conditional eddies in plasma turbulence. The Physics of Fluids, 30(7), 2239–2254. 10.1063/1.866158

[jgra57548-bib-0050] Karimabadi, H. , Daughton, W. , & Scudder, J. (2007). Multi‐scale structure of the electron diffusion region. Geophysical Research Letters, 34(13), L13104. 10.1029/2007gl030306

[jgra57548-bib-0051] Khotyaintsev, Y. V. , Vaivads, A. , André, M. , Fujimoto, M. , Retinò, A. , & Owen, C. J. (2010). Observations of slow electron holes at a magnetic reconnection site. Physical Review Letters, 105(16), 165002. 10.1103/physrevlett.105.165002 21230981

[jgra57548-bib-0052] Khrabrov, A. V. , & Sonnerup, B. U. Ö. (1998). deHoffmann‐Teller analysis. In G. Paschmann & P. W. Daly (Eds.), Analysis methods for multi‐spacecraft data (pp. 221–248). ESA Publication.

[jgra57548-bib-0053] Kliem, B. (1994). Particle orbits, trapping, and acceleration in a filamentary current sheet model. International Astronomical Union Colloquium, 142, 719–728. 10.1086/191896

[jgra57548-bib-0054] Krimigis, S. M. , & Sarris, E. T. (1980). Energetic particle bursts in the Earth’s magnetotail. In Paper presented at the dynamics of the magnetosphere.

[jgra57548-bib-0055] Li, X. , Guo, F. , Li, H. , & Li, G. (2017). Particle acceleration during magnetic reconnection in a low‐beta plasma. The Astrophysical Journal, 843(1), 21. 10.3847/1538-4357/aa745e

[jgra57548-bib-0056] Li, X. , Guo, F. , & Liu, Y.‐H. (2021). The acceleration of charged particles and formation of power‐law energy spectra in nonrelativistic magnetic reconnection. Physics of Plasmas, 28(5), 052905. 10.1063/5.0047644

[jgra57548-bib-0057] Liu, T. Z. , Lu, S. , Turner, D. L. , Gingell, I. , Angelopoulos, V. , Zhang, H. , et al. (2020). Magnetospheric multiscale (MMS) observations of magnetic reconnection in foreshock transients. Journal of Geophysical Research: Space Physics, 125(4), e2020JA027822. 10.1029/2020ja027822

[jgra57548-bib-0058] Lu, S. , Artemyev, A. V. , Angelopoulos, V. , & Pritchett, P. L. (2020). Energetic electron acceleration by ion‐scale magnetic islands in turbulent magnetic reconnection: Particle‐in‐cell simulations and ARTEMIS observations. The Astrophysical Journal, 896(2), 105. 10.3847/1538-4357/ab908e

[jgra57548-bib-0059] Matsumoto, H. , Deng, X. H. , Kojima, H. , & Anderson, R. R. (2003). Observation of electrostatic solitary waves associated with reconnection on the dayside magnetopause boundary. Geophysical Research Letters, 30(6), 1326. 10.1029/2002gl016319

[jgra57548-bib-0060] Nakamura, R. , Baumjohann, W. , Panov, E. , Volwerk, M. , Birn, J. , Artemyev, A. , et al. (2013). Flow bouncing and electron injection observed by Cluster. Journal of Geophysical Research: Space Physics, 118(5), 2055–2072. 10.1002/jgra.50134

[jgra57548-bib-0061] Northrop, T. G. (1963). Adiabatic charged‐particle motion. Reviews of Geophysics, 1(3), 283–304. 10.1029/rg001i003p00283

[jgra57548-bib-0062] Øieroset, M. , Lin, R. P. , Phan, T. D. , Larson, D. E. , & Bale, S. D. (2002). Evidence for electron acceleration up to ∼300 keV in the magnetic reconnection diffusion region of Earth's magnetotail. Physical Review Letters, 89(19), 195001. 10.1103/physrevlett.89.195001 12443119

[jgra57548-bib-0063] Oka, M. , Birn, J. , Battaglia, M. , Chaston, C. C. , Hatch, S. M. , Livadiotis, G. , et al. (2018). Electron power‐law spectra in solar and space plasmas. Space Science Reviews, 214(5), 82. 10.1007/s11214-018-0515-4

[jgra57548-bib-0064] Oka, M. , Phan, T. D. , Krucker, S. , Fujimoto, M. , & Shinohara, I. (2010). Electron acceleration by multi‐island coalescence. The Astrophysical Journal, 714(1), 915–926. 10.1088/0004-637x/714/1/915

[jgra57548-bib-0065] Oka, M. , Phan, T.‐D. , Øieroset, M. , & Angelopoulos, V. (2016). In situ evidence of electron energization in the electron diffusion region of magnetotail reconnection. Journal of Geophysical Research: Space Physics, 121(3), 1955–1968. 10.1002/2015ja022040

[jgra57548-bib-0066] Omura, Y. , Matsumoto, H. , Miyake, T. , & Kojima, H. (1996). Electron beam instabilities as generation mechanism of electrostatic solitary waves in the magnetotail. Journal of Geophysical Research, 101(A2), 2685–2697. 10.1029/95JA03145

[jgra57548-bib-0067] Paschmann, G. (1985). Comment on “electric field measurements at the magnetopause: 1, observation of large convective velocities at rotational magnetopause discontinuities” by T. L. Aggson, P. J. Gambardella, and N. C. Maynard. Journal of Geophysical Research, 90(A8), 7629–7630. 10.1029/JA090iA08p07629

[jgra57548-bib-0068] Pellinen, R. J. , & Heikkila, W. J. (1978). Energization of charged particles to high energies by an induced substorm electric field within the magnetotail. Journal of Geophysical Research, 83(A4), 1544–1550. 10.1029/ja083ia04p01544

[jgra57548-bib-0069] Phan, T. D. , Eastwood, J. P. , Shay, M. A. , Drake, J. F. , Sonnerup, B. U. Ö. , Fujimoto, M. , et al. (2018). Electron magnetic reconnection without ion coupling in Earth’s turbulent magnetosheath. Nature, 557(7704), 202–206. 10.1038/s41586-018-0091-5 29743689

[jgra57548-bib-0070] Pollock, C. , Moore, T. , Jacques, A. , Burch, J. , Gliese, U. , Saito, Y. , et al. (2016). Fast plasma investigation for magnetospheric multiscale. Space Science Reviews, 199(1), 331–406. 10.1007/s11214-016-0245-4

[jgra57548-bib-0071] Pritchett, P. L. (2008). Energetic electron acceleration during multi‐island coalescence. Physics of Plasmas, 15(10), 102105. 10.1063/1.2996321

[jgra57548-bib-0072] Propp, K. , & Beard, D. B. (1984). Cross‐tail ion drift in a realistic model magnetotail. Journal of Geophysical Research, 89(A12), 11013–11017. 10.1029/ja089ia12p11013

[jgra57548-bib-0073] Retinò, A. , Nakamura, R. , Vaivads, A. , Khotyaintsev, Y. , Hayakawa, T. , Tanaka, K. , et al. (2008). Cluster observations of energetic electrons and electromagnetic fields within a reconnecting thin current sheet in the Earth's magnetotail. Journal of Geophysical Research, 113(A12), A12215. 10.1029/2008ja013511

[jgra57548-bib-0074] Richardson, I. G. , Owen, C. J. , & Slavin, J. A. (1996). Energetic (>0.2 MeV) electron bursts in the deep geomagnetic tail observed by the Goddard Space Flight Center experiment on ISEE 3: Association with geomagnetic substorms. Journal of Geophysical Research, 101(A2), 2723–2740. 10.1029/95ja03375

[jgra57548-bib-0075] Russell, C. T. , Anderson, B. J. , Baumjohann, W. , Bromund, K. R. , Dearborn, D. , Fischer, D. , et al. (2016). The magnetospheric multiscale magnetometers. Space Science Reviews, 199(1), 189–256. 10.1007/s11214-014-0057-3

[jgra57548-bib-0076] Scholer, M. (1984). Energetic ions and electrons and their acceleration processes in the magnetotail. In E. W. Hones (Ed.), Magnetic reconnection in space and laboratory plasmas (pp. 216–227). American Geophysical Union.

[jgra57548-bib-0077] Scholer, M. , Klecker, B. , Hovestadt, D. , Gloeckler, G. , Ipavich, F. M. , & Galvin, A. B. (1985). Energetic particle characteristics of magnetotail flux ropes. Geophysical Research Letters, 12(4), 191–194. 10.1029/gl012i004p00191

[jgra57548-bib-0078] Sergeev, V. A. , Sazhina, E. M. , Tsyganenko, N. A. , Lundblad, J. Å. , & Søraas, F. (1983). Pitch‐angle scattering of energetic protons in the magnetotail current sheet as the dominant source of their isotropic precipitation into the nightside ionosphere. Planetary and Space Science, 31(10), 1147–1155. 10.1016/0032-0633(83)90103-4

[jgra57548-bib-0079] Shen, C. , Li, X. , Dunlop, M. , Shi, Q. Q. , Liu, Z. X. , Lucek, E. , & Chen, Z. Q. (2007). Magnetic field rotation analysis and the applications. Journal of Geophysical Research, 112(A6), A06211. 10.1029/2005ja011584

[jgra57548-bib-0080] Shi, Q. Q. , Shen, C. , Pu, Z. Y. , Dunlop, M. W. , Zong, Q. G. , Zhang, H. , et al. (2005). Dimensional analysis of observed structures using multipoint magnetic field measurements: Application to Cluster. Geophysical Research Letters, 32(12), L12105. 10.1029/2005gl022454

[jgra57548-bib-0081] Shi, Q. Q. , Tian, A. M. , Bai, S. C. , Hasegawa, H. , Degeling, A. W. , Pu, Z. Y. , et al. (2019). Dimensionality, coordinate system and reference frame for analysis of in‐situ space plasma and field data. Space Science Reviews, 215(4), 35. 10.1007/s11214-019-0601-2

[jgra57548-bib-0082] Slavin, J. A. , Baker, D. N. , Craven, J. D. , Elphic, R. C. , Fairfield, D. H. , Frank, L. A. , et al. (1989). CDAW 8 observations of plasmoid signatures in the geomagnetic tail: An assessment. Journal of Geophysical Research, 94(A11), 15153–15175. 10.1029/ja094ia11p15153

[jgra57548-bib-0083] Slavin, J. A. , Lepping, R. P. , & Baker, D. N. (1990). IMP‐8 observations of traveling compression regions: New evidence for near‐Earth plasmoids and neutral lines. Geophysical Research Letters, 17(7), 913–916. 10.1029/gl017i007p00913

[jgra57548-bib-0084] Slavin, J. A. , Lepping, R. P. , Gjerloev, J. , Fairfield, D. H. , Hesse, M. , Owen, C. J. , et al. (2003). Geotail observations of magnetic flux ropes in the plasma sheet. Journal of Geophysical Research, 108(A1), SMP 10‐11–SMP 10‐18. 10.1029/2002ja009557

[jgra57548-bib-0085] Slavin, J. A. , Smith, M. F. , Mazur, E. L. , Baker, D. N. , Iyemori, T. , Singer, H. J. , & Greenstadt, E. W. (1992). ISEE 3 plasmoid and TCR observations during an extended interval of substorm activity. Geophysical Research Letters, 19(8), 825–828. 10.1029/92gl00394

[jgra57548-bib-0086] Sonnerup, B. U. Ö. , & Guo, M. (1996). Magnetopause transects. Geophysical Research Letters, 23(25), 3679–3682. 10.1029/96gl03573

[jgra57548-bib-0087] Sturrock, P. A. (1994). Plasma physics: An introduction to the theory of astrophysical, geophysical and laboratory plasmas. Cambridge University Press.

[jgra57548-bib-0088] Sun, W. J. , Slavin, J. A. , Dewey, R. M. , Raines, J. M. , Fu, S. Y. , Wei, Y. , et al. (2018). A comparative study of the proton properties of magnetospheric substorms at Earth and mercury in the near magnetotail. Geophysical Research Letters, 45(16), 7933–7941. 10.1029/2018gl079181

[jgra57548-bib-0089] Sun, W. J. , Slavin, J. A. , Tian, A. M. , Bai, S. C. , Poh, G. K. , Akhavan‐Tafti, M. , et al. (2019). MMS study of the structure of ion‐scale flux ropes in the Earth's cross‐tail current sheet. Geophysical Research Letters, 46(12), 6168–6177. 10.1029/2019gl083301

[jgra57548-bib-0090] Torbert, R. B. , Russell, C. T. , Magnes, W. , Ergun, R. E. , Lindqvist, P. A. , LeContel, O. , et al. (2016). The FIELDS instrument suite on MMS: Scientific objectives, measurements, and data products. Space Science Reviews, 199(1), 105–135. 10.1007/s11214-014-0109-8

[jgra57548-bib-0091] Turner, D. L. , Cohen, I. J. , Bingham, S. T. , Stephens, G. K. , Sitnov, M. I. , Mauk, B. H. , et al. (2021). Characteristics of energetic electrons near active magnetotail reconnection sites: Tracers of a complex magnetic topology and evidence of localized acceleration. Geophysical Research Letters, 48(2), e2020GL090089. 10.1029/2020gl090089

[jgra57548-bib-0092] Wang, R. , Lu, Q. , Du, A. , & Wang, S. (2010). In situ observations of a secondary magnetic island in an ion diffusion region and associated energetic electrons. Physical Review Letters, 104(17), 175003. 10.1103/physrevlett.104.175003 20482115

[jgra57548-bib-0093] Wang, R. , Vasko, I. Y. , Artemyev, A. V. , Holley, L. C. , Kamaletdinov, S. R. , Lotekar, A. , & Mozer, F. S. (2022). Multisatellite observations of ion holes in the Earth's plasma sheet. Geophysical Research Letters, 49(8), e2022GL097919. 10.1029/2022GL097919

[jgra57548-bib-0094] Wang, S. , Chen, L.‐J. , Bessho, N. , Kistler, L. M. , Shuster, J. R. , & Guo, R. (2016). Electron heating in the exhaust of magnetic reconnection with negligible guide field. Journal of Geophysical Research: Space Physics, 121(3), 2104–2130. 10.1002/2015ja021892

[jgra57548-bib-0095] Wetherton, B. A. , Egedal, J. , Le, A. , & Daughton, W. (2021). Anisotropic electron fluid closure validated by in situ spacecraft observations in the far exhaust of guide‐field reconnection. Journal of Geophysical Research: Space Physics, 126(1), e2020JA028604. 10.1029/2020JA028604

[jgra57548-bib-0096] Wu, M. , Huang, C. , Lu, Q. , Volwerk, M. , Nakamura, R. , Vörös, Z. , et al. (2015). In situ observations of multistage electron acceleration driven by magnetic reconnection. Journal of Geophysical Research: Space Physics, 120(8), 6320–6331. 10.1002/2015ja021165

[jgra57548-bib-0097] Xiong, Q. Y. , Huang, S. Y. , Zhou, M. , Yuan, Z. G. , Deng, X. H. , Jiang, K. , et al. (2022). Formation of negative J ⋅ E′ in the outer electron diffusion region during magnetic reconnection. Journal of Geophysical Research: Space Physics, 127(2), e2022JA030264. 10.1029/2022ja030264

[jgra57548-bib-0098] Xu, Y. , Fu, H. S. , Liu, C. M. , & Wang, T. Y. (2018). Electron acceleration by dipolarization fronts and magnetic reconnection: A quantitative comparison. The Astrophysical Journal, 853(1), 11. 10.3847/1538-4357/aa9f2f

[jgra57548-bib-0099] Yamada, M. , Kulsrud, R. , & Ji, H. (2010). Magnetic reconnection. Reviews of Modern Physics, 82(1), 603–664. 10.1103/revmodphys.82.603

[jgra57548-bib-0100] Young, D. T. , Burch, J. L. , Gomez, R. G. , De Los Santos, A. , Miller, G. P. , Wilson, P. , et al. (2016). Hot plasma composition analyzer for the magnetospheric multiscale mission. Space Science Reviews, 199(1), 407–470. 10.1007/s11214-014-0119-6

[jgra57548-bib-0101] Young, S. L. , Denton, R. E. , Anderson, B. J. , & Hudson, M. K. (2008). Magnetic field line curvature induced pitch angle diffusion in the inner magnetosphere. Journal of Geophysical Research, 113(A3), A03210. 10.1029/2006ja012133

[jgra57548-bib-0102] Zenitani, S. , Hesse, M. , Klimas, A. , & Kuznetsova, M. (2011). New measure of the dissipation region in collisionless magnetic reconnection. Physical Review Letters, 106(19), 195003. 10.1103/physrevlett.106.195003 21668168

[jgra57548-bib-0103] Zhang, Q. , Drake, J. F. , & Swisdak, M. (2019a). Instabilities and turbulence in low‐β guide field reconnection exhausts with kinetic Riemann simulations. Physics of Plasmas, 26(10), 102115. 10.1063/1.5121782

[jgra57548-bib-0104] Zhang, Q. , Drake, J. F. , & Swisdak, M. (2019b). Particle heating and energy partition in low‐β guide field reconnection with kinetic Riemann simulations. Physics of Plasmas, 26(7), 072115. 10.1063/1.5104352

[jgra57548-bib-0105] Zhang, Q. , Guo, F. , Daughton, W. , Li, H. , & Li, X. (2021). Efficient nonthermal ion and electron acceleration enabled by the flux‐rope kink instability in 3D nonrelativistic magnetic reconnection. Physical Review Letters, 127(18), 185101. 10.1103/physrevlett.127.185101 34767407

[jgra57548-bib-0106] Zhang, Y. C. , Shen, C. , Liu, Z. X. , Rong, Z. J. , Zhang, T. L. , Marchaudon, A. , et al. (2013). Two different types of plasmoids in the plasma sheet: Cluster multisatellite analysis application. Journal of Geophysical Research: Space Physics, 118(9), 5437–5444. 10.1002/jgra.50542

[jgra57548-bib-0107] Zhong, Z. H. , Zhou, M. , Tang, R. X. , Deng, X. H. , Turner, D. L. , Cohen, I. J. , et al. (2020). Direct evidence for electron acceleration within ion‐scale flux rope. Geophysical Research Letters, 47(1), e2019GL085141. 10.1029/2019gl085141

[jgra57548-bib-0108] Zhou, M. , Li, T. , Deng, X. , Pang, Y. , Xu, X. , Tang, R. , et al. (2016). Statistics of energetic electrons in the magnetotail reconnection. Journal of Geophysical Research: Space Physics, 121(4), 3108–3119. 10.1002/2015ja022085

[jgra57548-bib-0109] Zhou, M. , Man, H. Y. , Deng, X. H. , Pang, Y. , Khotyaintsev, Y. , Lapenta, G. , et al. (2021). Observations of secondary magnetic reconnection in the turbulent reconnection outflow. Geophysical Research Letters, 48(4), e2020GL091215. 10.1029/2020gl091215

[jgra57548-bib-0110] Zong, Q. G. , Fritz, T. A. , Spence, H. , Oksavik, K. , Pu, Z. Y. , Korth, A. , & Daly, P. W. (2004). Energetic particle sounding of the magnetopause: A contribution by cluster/RAPID. Journal of Geophysical Research, 109(A4), A04207. 10.1029/2003ja009929

[jgra57548-bib-0111] Zong, Q.‐G. , Wilken, B. , Reeves, G. D. , Daglis, I. A. , Doke, T. , Iyemori, T. , et al. (1997). Geotail observations of energetic ion species and magnetic field in plasmoid‐like structures in the course of an isolated substorm event. Journal of Geophysical Research, 102(A6), 11409–11428. 10.1029/97ja00076

[jgra57548-bib-0112] Zong, Q.‐G. , & Zhang, H. (2018). In situ detection of the electron diffusion region of collisionless magnetic reconnection at the high‐latitude magnetopause. Earth and Planetary Physics, 2(3), 231–237.

[jgra57548-bib-0113] Zweibel, E. G. , & Yamada, M. (2009). Magnetic reconnection in astrophysical and laboratory plasmas. Annual Review of Astronomy and Astrophysics, 47(1), 291–332. 10.1146/annurev-astro-082708-101726

