# Supplementary material for: Properties and Acceleration Mechanisms of Electrons Up To 200 keV Associated With a Flux Rope Pair and Reconnection X‐Lines Around It in Earth's Plasma Sheet
Source: J Geophys Res Space Phys. 2022 Dec 23;127(12):e2022JA030721. doi: 10.1029/2022JA030721 (PMC10078532; doi:10.1029/2022JA030721)
Supplement: Supplementary file 1 — Supporting Information S1 [file JGRA-127-0-s001.docx]

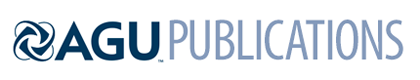


*Journal of Geophysical Research – Space Physics*

Supporting Information for

**Properties and acceleration mechanisms of electrons up to 200 keV associated with a flux rope pair and reconnection X-lines in Earth’s plasma sheet**

Weijie Sun^1^, Drew L. Turner^2^, Qile Zhang^3^, Shan Wang^4^, Jan. Egedal^5^, Trevor Leonard^6^,

James A. Slavin^1^, Qiang Hu^7^, Ian J Cohen^2^, Kevin Genestreti^8^, Gangkai Poh^9,10^,

Daniel J. Gershman^9^, Andrew Smith^11,12^, Guan Le^9^, Rumi Nakamura^13^, Barbara L. Giles^9^,

Robert E. Ergun^14^, and Jim L. Burch^8^

^1^, Department of Climate and Space Sciences and Engineering, University of Michigan, Ann Arbor, MI 48109, USA

^2^, Space Exploration Sector, Johns Hopkins Applied Physics Laboratory, Laurel, MD, USA

^3^, Los Alamos National Laboratory, Los Alamos, NM, USA

^4^, Department of Astronomy, University of Maryland, College Park, MD, USA

^5^, Department of Physics, University of Wisconsin-Madison, Madison, WI, USA

^6^, Laboratory for Atmospheric and Space Physics, University of Colorado Boulder, CO, USA

^7^, Department of Space Science, and Center for Space Plasma and Aeronomic Research, the University of Alabama in Huntsville, Huntsville, AL, USA

^8^, Southwest Research Institute, San Antonio, TX, USA

^9^, NASA Goddard Space Flight Center, Greenbelt, MD, USA

^10^, Center for Research and Exploration in Space Sciences and Technology II, Catholic University of America, Washington, DC, USA

^11^, Mullard Space Science Laboratory, University College London, Dorking, Surrey, UK

^12^, Department of Mathematics, Physics and Electrical Engineering, Northumbria University, Newcastle upon Tyne, UK

^13^, Space Research Institute, Austrian Academy of Sciences, Graz, Austria

^14^, Department of Astrophysical and Planetary Sciences, University of Colorado Boulder, Boulder, CO, USA

Correspondence to [wjsun@umich.edu](mailto:wjsun@umich.edu)

**Introduction**

This file provides supporting information for the above paper. We provide:

Figure S1. Overview of the measurements from MMS1 on 12 July 2017 between 14:00 to 15:00 UTC.

Figure S2. Scatter plot of the data points of the Betatron term obtained from the electron bulk velocity measured by FPI and the drifting velocity due to convection electric field

Figure S3. Scatter plot of the data points of the Fermi term obtained from the electron bulk velocity measured by FPI and the drifting velocity due to convection electric field.

Figure S4. The comparisons of the current density between the curlometer and particles.


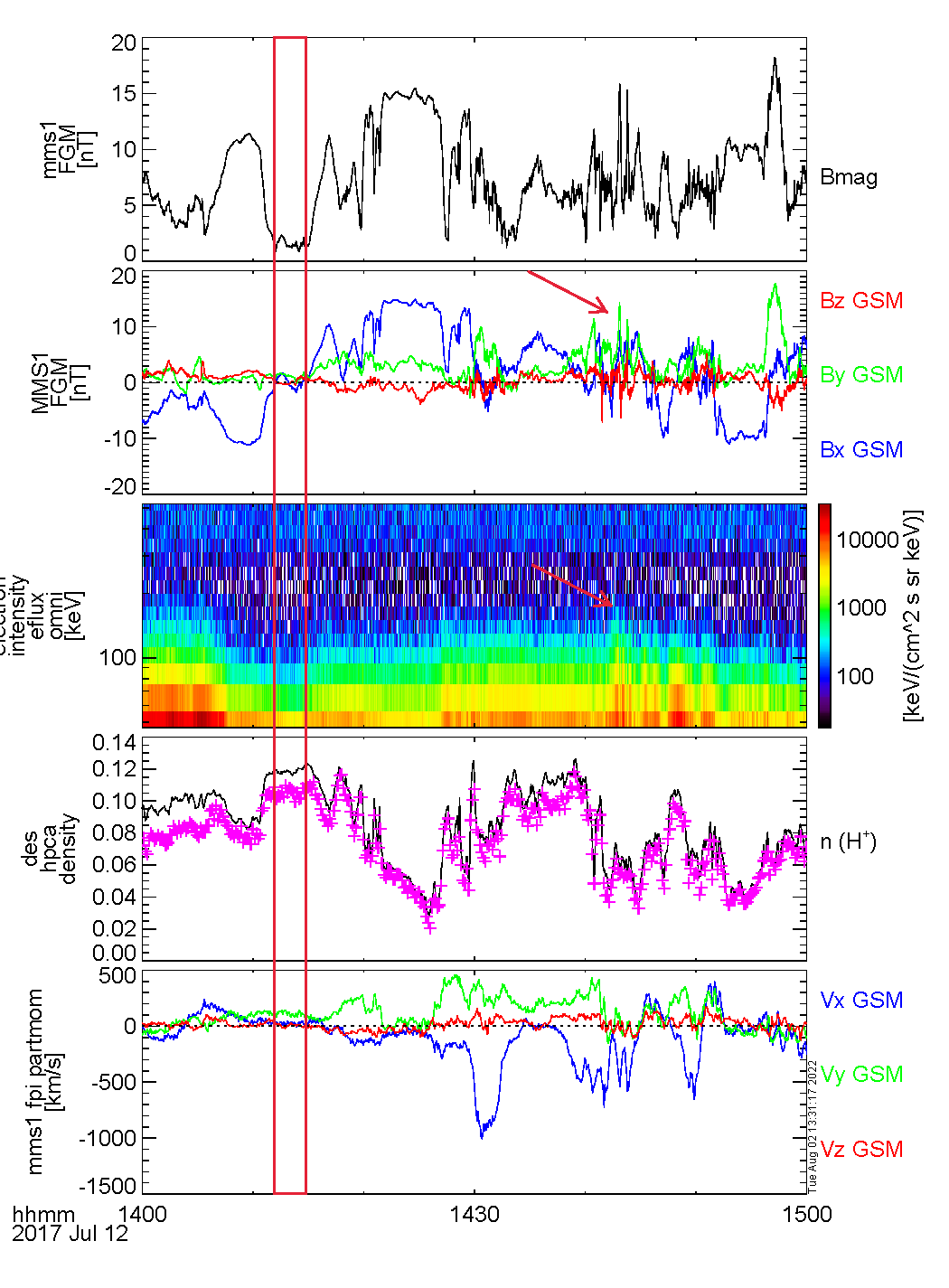


Figure S1. Overview of the measurements from MMS1 on 12 July 2017 between 14:00 to 15:00 UTC. From top to bottom: magnetic field intensity; magnetic field vectors in GSM coordinate, B_x_ in blue, B_y_ in green, B_z_ in red; energetic-electron spectrum from FEEPS (> 47.2 keV); electron umber density measured by FPI (solid line) and ion number density measured by HPCA (crosses); Ion bulk velocity measured by FPI, V_x_ in blue, V_y_ in green, V_z_ in red.


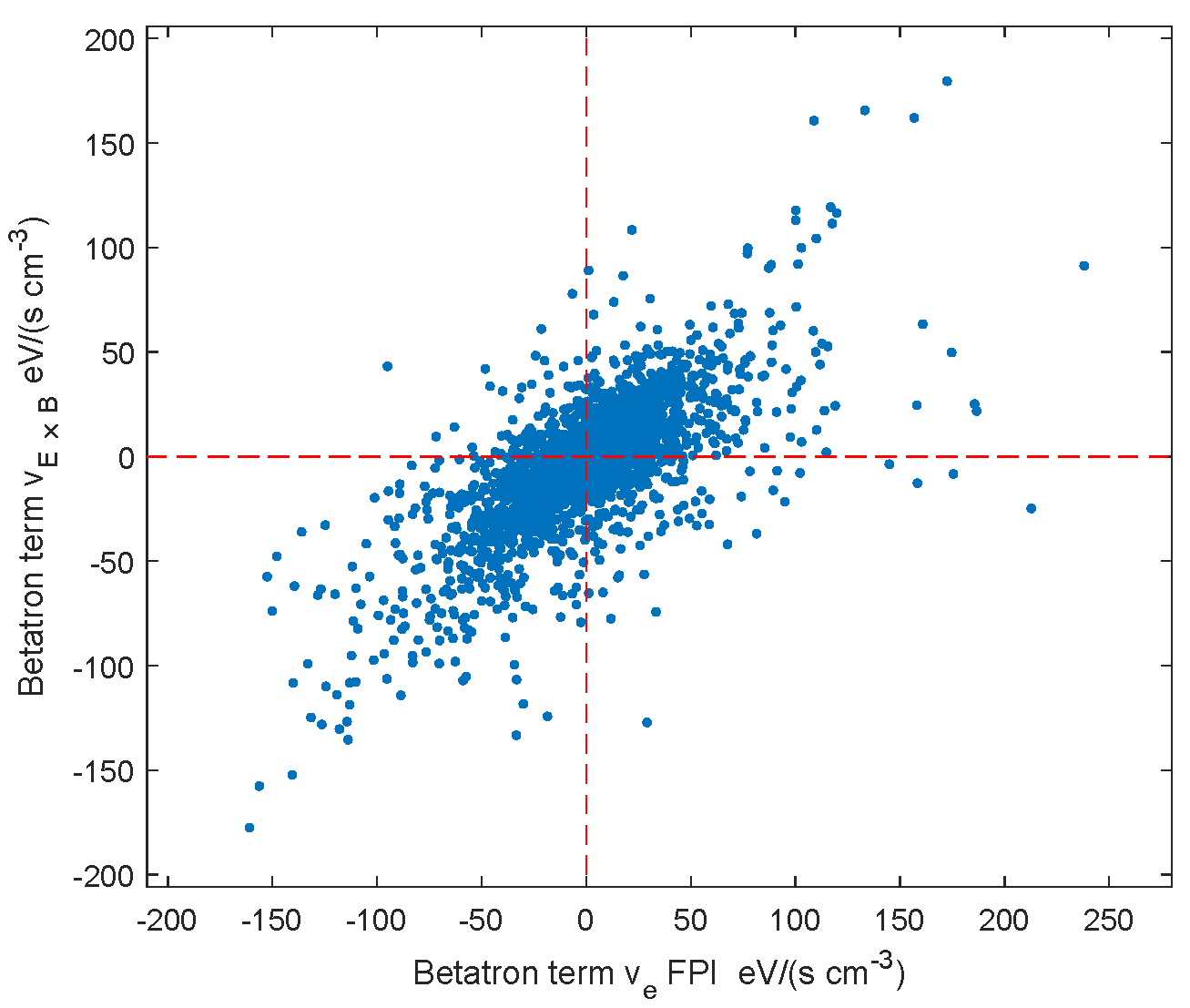


Figure S2. Scatter plot of the data points of the Betatron term obtained from the electron bulk velocity measured by FPI and the drifting velocity due to convection electric field.


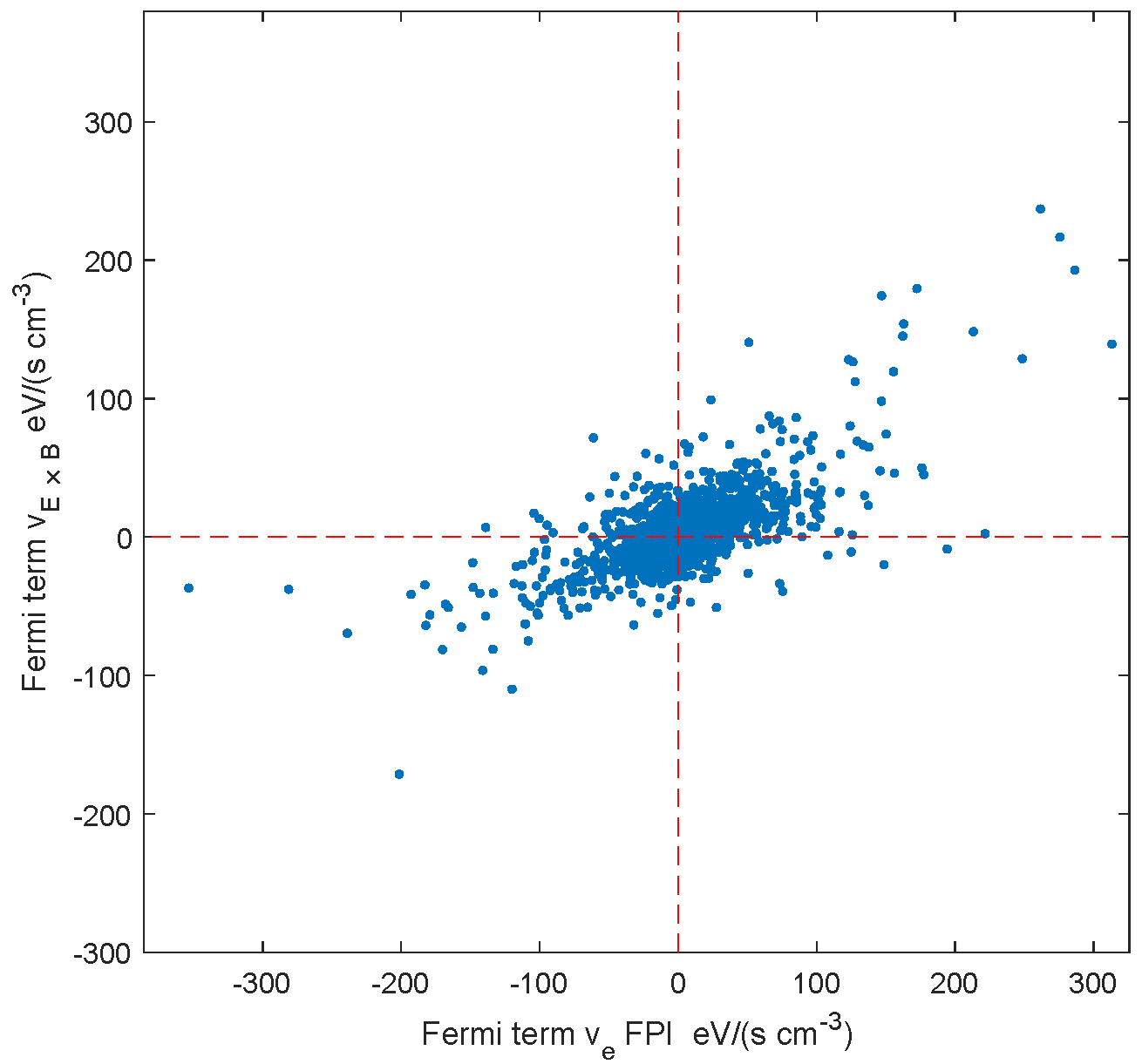


Figure S3. Scatter plot of the data points of the Fermi term obtained from the electron bulk velocity measured by FPI and the drifting velocity due to convection electric field.


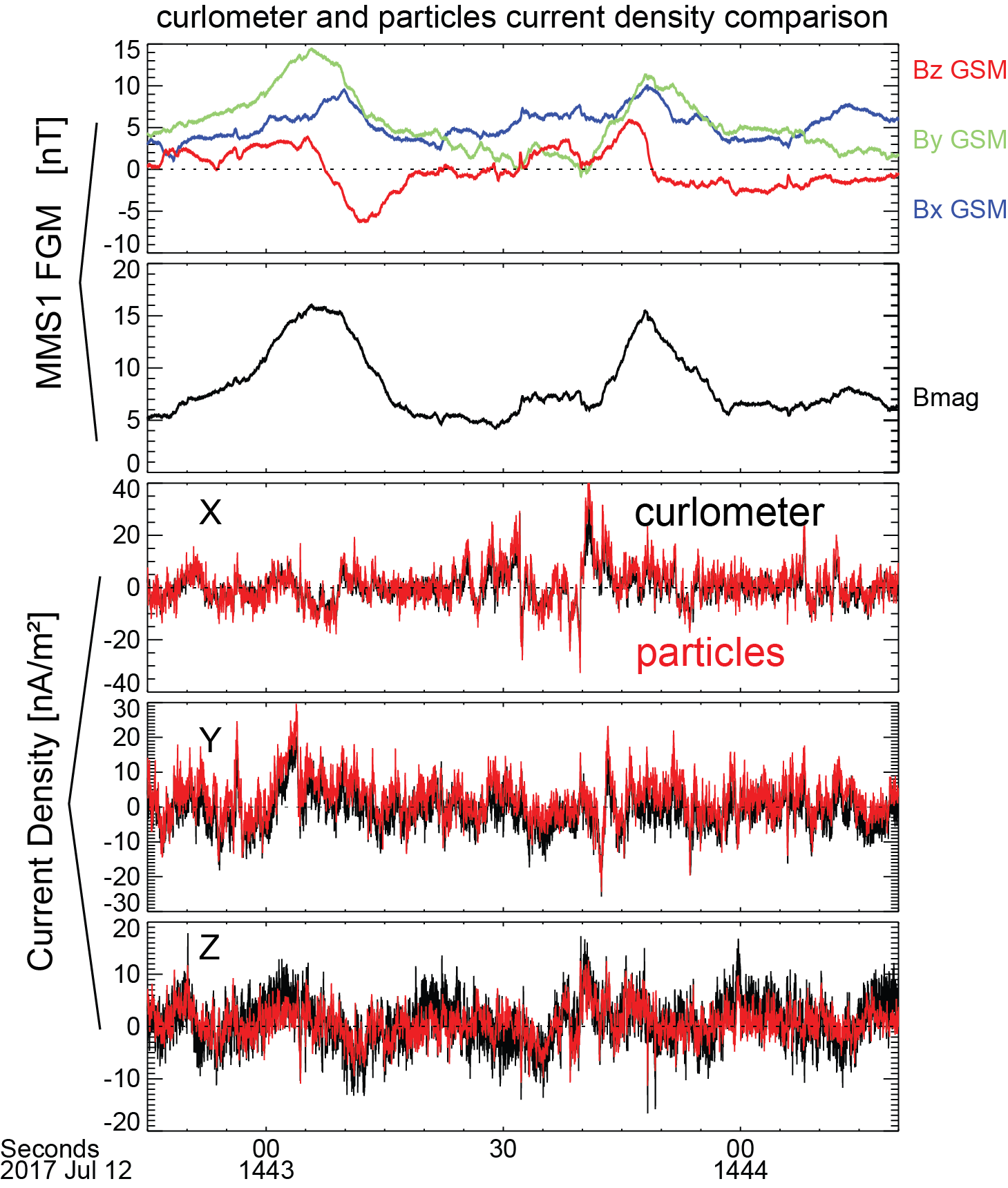


Figure S4. The comparisons of the current density between the curlometer and particles.
